# Identification of Immune-Related lncRNA Regulatory Network in Pulpitis

**DOI:** 10.1155/2022/7222092

**Published:** 2022-06-06

**Authors:** Jing Du, Liu Liu, Fan Yang, Sha Leng, Lan Zhang, Dingming Huang

**Affiliations:** ^1^State Key Laboratory of Oral Diseases & National Clinical Research Center for Oral Diseases, West China Hospital of Stomatology, Sichuan University, Chengdu 610041, China; ^2^Department of Conservative Dentistry and Endodontics, West China Hospital of Stomatology, Sichuan University, Chengdu 610041, China

## Abstract

**Background:**

Long noncoding RNAs (lncRNAs) are emerging as critical regulators of various biological processes, including immune regulation.

**Methods:**

Due to the critical significance of immunological responses in the development and progression of pulpitis, we used an integrated algorithm to identify immune-related lncRNAs and then examined the lncRNA-immunity regulation network in pulpitis. Before identifying immune-related lncRNAs, the data from GEO datasets were precleaned. ConsensusClusterPlus was used to differentiate immune-related pulpitis subgroups. Enrichment analysis using GO and MSigDB databases was employed to determine the differences in molecular function, cellular component, and biological process between the two pulpitis subtypes.

**Results:**

An integrated algorithm was designed to filtrate immune-related lncRNAs accurately. 790 immune-related lncRNAs were found in 17 immunological categories, with 38 of them perturbated in pulpitis. The Cytoscape software was used to visualize the relationship between representative immune regulatory pathways and immune-related lncRNAs. Two immune-related pulpitis subtypes were discovered using differentially expressed immune-related lncRNAs. Subtype 2 has a stronger association with immune-related pathways than subtype 1 does.

**Conclusions:**

Our study identified many immune-related lncRNAs and investigated potential lncRNA regulation networks; meanwhile, the molecular subtypes of pulpitis were identified, all of which will be relevant for further research into inflammatory and immunological processes in pulpitis.

## 1. Introduction

Pulpitis is a typical inflammatory response that occurs in the pulp tissue. Caries cause demineralization of the enamel and dental defects. If suitable treatments are not taken, caries will eventually progress to pulp infection and necrosis. Within the pulp, a complex biochemical process occurs in response to the attack of bacteria's DNA, lipopolysaccharides (LPSs), and lipoteichoic acids (LTAs) [1]. When triggered, microbial ligands interact with pattern recognition receptors (PRRs) and activate the nuclear factor kappa B (NF-*κ*B) and mitogen-activated protein kinase (MAPK) pathways [2, 3]. Fibroblasts, odontoblasts, T lymphocytes, and mesenchymal cells in the dental pulp secrete a variety of inflammatory mediators, including cytokines and chemokines, which play a critical role in the pulp's response to stimuli [4]. As a result, therapy regimens that aim to reduce cytokine and chemokine levels and activity are advantageous. Recent pulpitis research has concentrated on the molecular mechanisms through which inflammatory mediators regulate the onset and course of pulpitis and specific regulatory networks. Numerous cellular and molecular level researches have focused on pulpitis indicators; however, they have primarily used low-throughput methods and highlighted only a few molecular markers [5, 6]. Recently, more evidence showed that the currently used diagnostic term “irreversible pulpitis” is not accurate since extensively inflamed pulp can be saved by appropriate intervention. This has proven to be successful, and teeth diagnosed with irreversible pulpitis have been successfully treated with a pulpotomy [7, 8]. Recently, a finer classification [9] was proposed to reclassify the pulpitis into four stages (initial, mild, moderate, and severe pulpitis), but its molecular basis, especially at a high-throughput level, is still insufficient.

Noncoding RNAs are well-known modulators of the inflammatory response in the dental pulp. Long noncoding RNAs (lncRNAs) are a class of noncoding RNAs that exceed 200 nucleotides in length [10]. According to accumulating evidence, lncRNAs are involved in various biological processes, including transcription interference, mRNA stability, protein localization, and intranuclear trafficking [11]. They participate in various activities, including immune responses, tumor development, aging, and cell proliferation and differentiation [12, 13]. Recently, a small number of experimental studies and bioinformatics analyses revealed new information about the function of lncRNAs in pulpitis [14–18]. Huang and Chen screened for differentially expressed lncRNAs between normal and inflamed pulp tissue using an integrated comparative technique. The results suggested that lncRNAs played a significant role in pulpitis pathogenesis and development [18]. MEG3 long noncoding RNA has been demonstrated as a negative regulator in the pulpitis [15]. Additionally, Wang et al. established that NUTM2A-AS1 was required for the development and progression of pulpitis [19].

Although a few studies have reported the effect of lncRNAs on pulpitis, the regulatory network underlying inflammatory responses remains unknown. Our study employed bioinformatic techniques to identify immune-related lncRNAs associated with pulpitis. In pulpitis, we observed immune-related lncRNA perturbation and activation of inflammatory pathways, as well as a novel classification of pulpitis based on the immune-related lncRNAs. These findings suggest a new direction for further research into the involvement of lncRNAs in pulpitis, potentially aiding in its diagnostic classification and therapy.

## 2. Materials and Methods

### 2.1. Data Acquisition and Processing

GSE92681 and GSE77459 from the Gene Expression Omnibus (GEO) datasets provided the expression profiling. GSE92681 contained 5 healthy pulp tissue samples and 7 inflamed pulp tissue samples, while GSE77459 contained 6 pulpitis tissue specimens and 6 normal pulp tissue specimens. According to the American Association of Endodontists (AAE), the inflamed pulp tissue was obtained entirely from teeth diagnosed with irreversible pulpitis.  Table 1 provides specific information about the samples. The immune gene list, which included 17 immunological categories, was downloaded via the ImmPort portal. The precleaning procedures of the gene expression matrix were the same as those in our previous study [20].

### 2.2. Identification of Immune-Related DElncRNAs in Pulpitis

The Linear Models for Microarray Data (limma) package in R software was used to examine differentially expressed mRNAs (DEmRNAs) and long non-coding RNAs (DElncRNAs) between normal and inflamed pulp tissue. Then, the connections between expression data of DEmRNAs and DElncRNAs were correlated and integrated by a computational approach. Simply put, the expression correlation between mRNA and lncRNA was discovered, and each lncRNA was corresponded to a relational table of mRNAs. These mRNAs were ranked according to the correlation of lncRNA-mRNA pairs. The immune-related gene set was then identified to see if it enriched at the top or bottom of the sorted mRNA list.

To be more explicit, each lncRNA-mRNA pair has its correlation coefficient (CCi) and *P* value (Pi) calculated using the R package psych. The rank score (RS) was determined using the following formula: RS = −log10(Pi)∗sign (CCi). All mRNAs were ranked by their corresponding RS scores. Following that, 17 immune-related categories were mapped to the ranked mRNA list using GSEA function of the R package clusterProfiler under default parameters, and the lncRNA-related enrichment score (lncRES) was calculated using enrichment score (ES) and *P* value obtained from GESA analysis [21]. The formula is

(1) $$\begin{array}{c} \text{lncRES }\left(\text{ik}\right)=\left\{\begin{array}{c} 1-2p,\text{ES}\left(\text{ik}\right)>0,\\ 2p-1,\text{ES}\left(\text{ik}\right)<0. \end{array}\right. \end{array}$$

The ES (ik) refers to the enrichment score between lncRNA (i) and immune-related gene-set (k). lncRNA-mRNA pairs with lncRES score > 0.995 and false discovery rate (FDR) < 0.05 were considered as the significant ones.

### 2.3. Functional Enrichment Analysis of Immune-Related lncRNAs

To investigate the pathways and biological processes involved in immune-related lncRNAs, we integrated the ranked mRNA list and hallmark pathways to GSEA analysis. The relationship between immune-related lncRNAs and 5 representative pathways was demonstrated using the software Cytoscape.

### 2.4. Classification of Pulpitis Patients Based on Immune-Related lncRNAs

The R package ConsensusClusterPlus (parameters: reps = 1000, pItem = 0.8, pFeature = 1, distance = euclidean) was used for discovering molecular subtypes based on the expression level of immune-related lncRNA. To obtain a stable cluster, the samples were separated into two groups. Principal component analysis (PCA) was used to evaluate the differences between the two clusters. The molecular function, cellular component, and pathway analyses were performed using Gene Ontology (GO) and hallmark gene sets in the Molecular Signature Database (MSigDB) separately. *P* value < 0.05 was reckoned statistically significant. Gene set variation analysis (GSVA) was conducted using gsva function in clusterProfiler. The decomposition of the cellular component was conducted using the R package Microenvironment Cell Populations-counter (MCPcounter) using its default probe sets and gene expression matrix [22].

### 2.5. Quantitative Real-Time PCR (qRT-PCR) Validation of Selected lncRNAs

DElncRNAs and immune-related lncRNAs were combined to produce 38 immune-related DElncRNAs, as previously stated. We used TRIzol Reagent (Invitrogen, USA) to extract total RNA from normal and inflamed pulp tissue to validate the identified lncRNAs. The inclusion criteria of samples referred to the published literature [20]. The detailed information of patients is shown in Table 2. cDNA was synthesized using PrimeScript RT reagent Kit gDNA Eraser (Takara, Japan). qRT-PCR was performed in 20 *μ*L volumes with SYBR Green PCR Master Mix (Roche, USA). The run methods are 95°C for 30 s and 40 cycles at 95°C for 5 s and 60°C for 34 s using QuantStudio Real-Time PCR Systems (Thermo Fishier, USA). By normalizing to the endogenous reference GAPDH, the expression of target genes was estimated. These experiments were approved by the Ethics Committee of West China Hospital of Stomatology, Sichuan University (approval number: WCHSIRB-d-2020-368). The experiments were in complianced with the Minimum Information for the Publication of Real-Time Quantitative PCR Experiments (MIQE) guidelines [23] (Table S1).

### 2.6. Workflow of the Study

**Figure 1:**
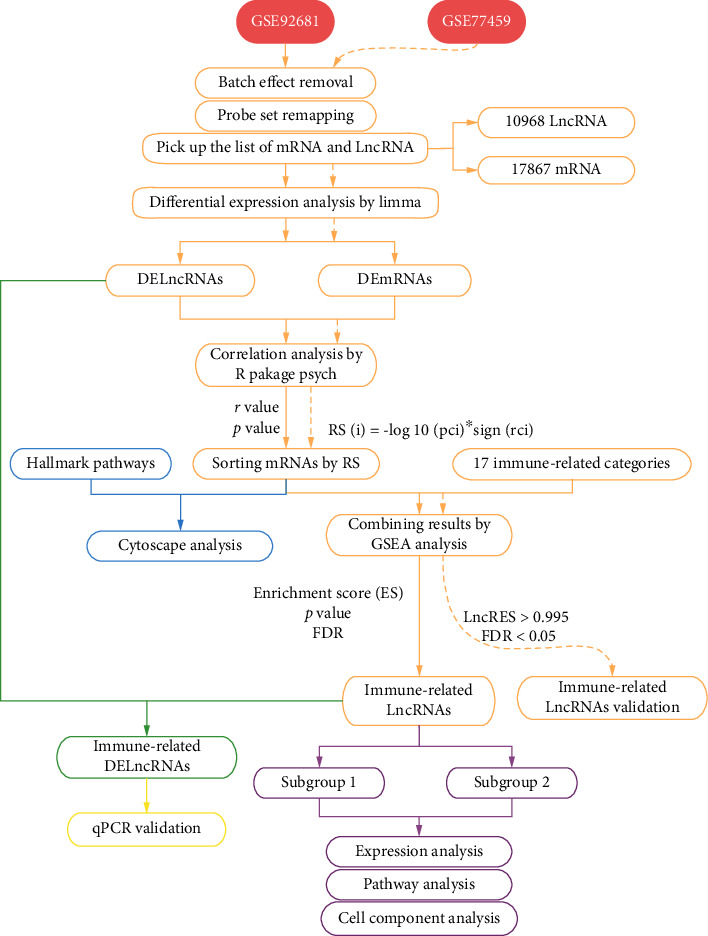
The workflow of this study.

To visualize our research approach intuitively, we illustrated a flow chart, which is depicted in Figure 1.

## 3. Results

### 3.1. Identification of Immune-Related lncRNAs in Pulpitis

**Figure 2:**
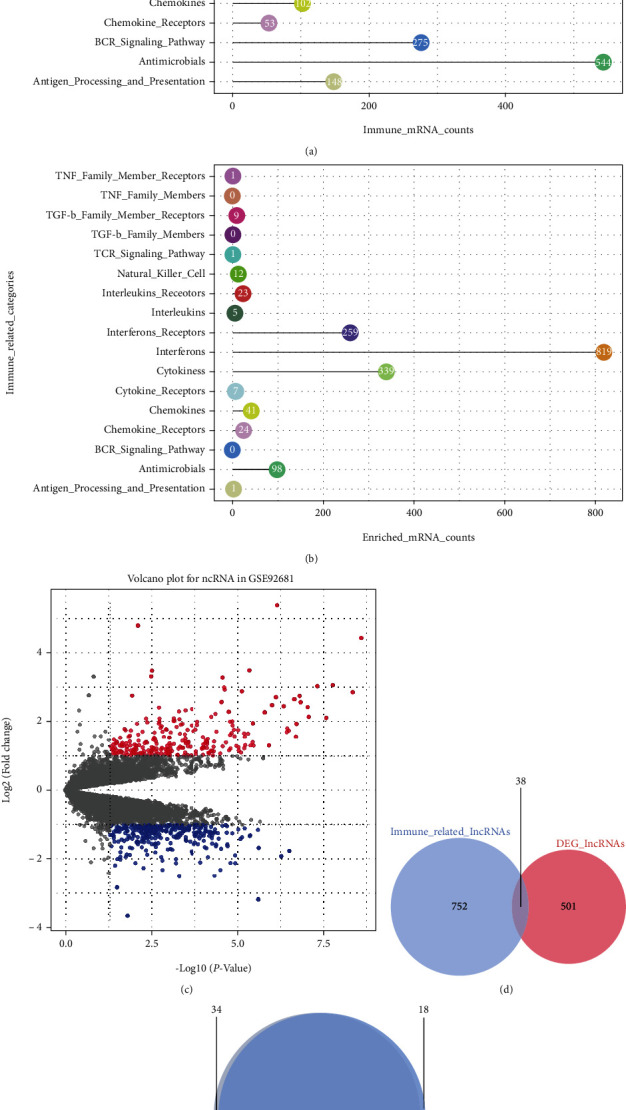
Finding immune-related lncRNAs in pulpitis. (a) 17 categories of immune-related genes. (b) Distribution of immune-related lncRNAs in 17 categories. (c) Volcano plot of DElncRNAs between normal and inflamed pulp tissue. Red dots: upregulated, green dots: downregulated, and gray dots: not statistic differences. (d) 38 lncRNAs were identified as immune-related DElncRNAs. (e) The highly overlap of two independent sets with the algorithm.

A range of data processing procedures was used to clean and integrate the data sets. As shown in Fig. S1and Fig. S2, sound normalization was obtained, and 10698 lncRNAs and 17867 mRNAs were picked up separately. 17 immune-related gene sets originating from distinct immunological pathways were retrieved using the Immunology Database and Analysis Portal (ImmPort). The categories of immunologically important genes are depicted in Figure 2(a). Over 85% of immune-related lncRNAs were discovered in the categories of interferons, interferon receptors, and cytokines, most notably the interferons and interferon receptor pathways, which were predicted to be potential targets for pulpitis treatment (Figure 2(b)). Differentially expressed lncRNAs and mRNAs were observed between normal and pulpitis samples (Figure 2(c) and Fig. S3). Our study's screening of lncRNA modulators provided insight into the immunologic pathways driving pulpitis.

### 3.2. Expression Perturbation and Validation of Immune-Related lncRNA Regulators

We then examined the correlation between selected DEmRNAs and DElncRNAs to understand the detailed function of immune-related lncRNAs better. We obtained 10698 relational tables, each of which had 17867 mRNAs and then sequenced these mRNAs according to their ranking score (RS). 539 DElncRNAs and 790 immune-related lncRNAs were identified, with 38 lncRNAs belonging to both (Figure 2(d)). Table S2 contains pertinent information on these lncRNAs. To validate the findings filtered out by our technique, we applied it to another dataset, GSE77459; the results indicated that the data from the two sets coincide virtually identically (Figure 2(e)). As a result, our approach was able to detect immune-related lncRNAs reliably.

### 3.3. Construction of Immune-Related lncRNAs and Hallmark Pathway Regulatory Network

**Figure 3:**
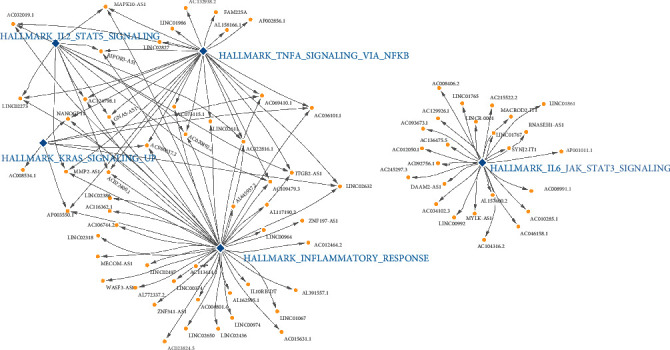
The relationship between immune-related lncRNAs and the representative hallmark pathways as seen using the software Cytoscape.

To elucidate the molecular mechanisms underlying the production of immune-related lncRNAs, we examined the relationship between immune-related lncRNAs and MSigDB hallmark pathways. The enriched lncRNAs and associated hallmark pathways are documented in Table S3, and the intuitive linkage of certain sample pathways relevant to vaccination and immune-related lncRNAs was demonstrated using Cytoscape software (Figure 3).

### 3.4. Immune-Related lncRNAs Classify Molecular Subtypes of Pulpitis

**Figure 4:**
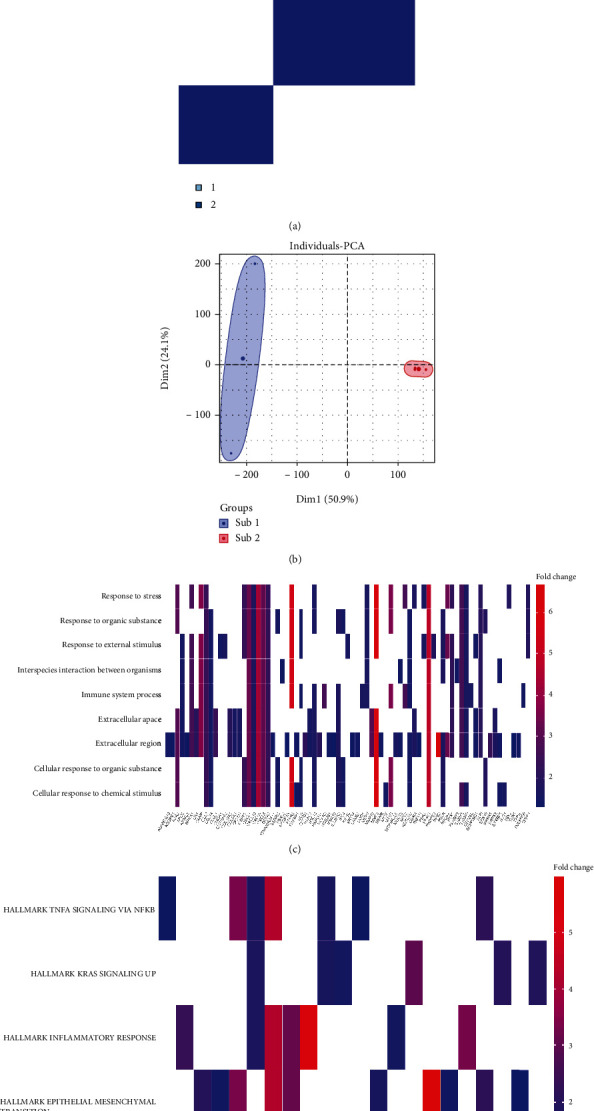
Subclustering analysis of pulpitis datasets. (a) Consensus clustering analysis of the immune-related lncRNAs, consensus matrix *K* = 2. (b) PCA of 38 perturbed immune-related lncRNAs between two pulpitis immune subtypes. The immune subtypes of the top 50 DEmRNAs (c) and DElncRNAs (d) displaying by cluster heatmaps.

To categorize the molecular subtypes of pulpitis, immune-related lncRNAs were found. Consensus clustering analysis was performed to divide pulpitis into two subgroups (Figure 4(a)). To validate the viability of the consensus clustering analysis, which used the characteristics of immune-related lncRNAs as classification criteria, the variance between samples was assessed by PCA. As illustrated in Figure 4(b), the distribution patterns of the two groups were significantly different, with the evenly inner-group distribution of clusters 1 and 2, showing that the consensus clustering analysis classification was reliable. To better understand the distribution and function of DEmRNAs and DElncRNAs in two pulpitis subgroups, we visualized the top 50 DEmRNAs and DElncRNAs with the lowest *P* values using hierarchical clustering heatmaps (Figures 4(c) and 4(d)). The heatmaps are shown in Fig S4.

### 3.5. Immune Characteristics of Pulpitis Subgroups

**Figure 5:**
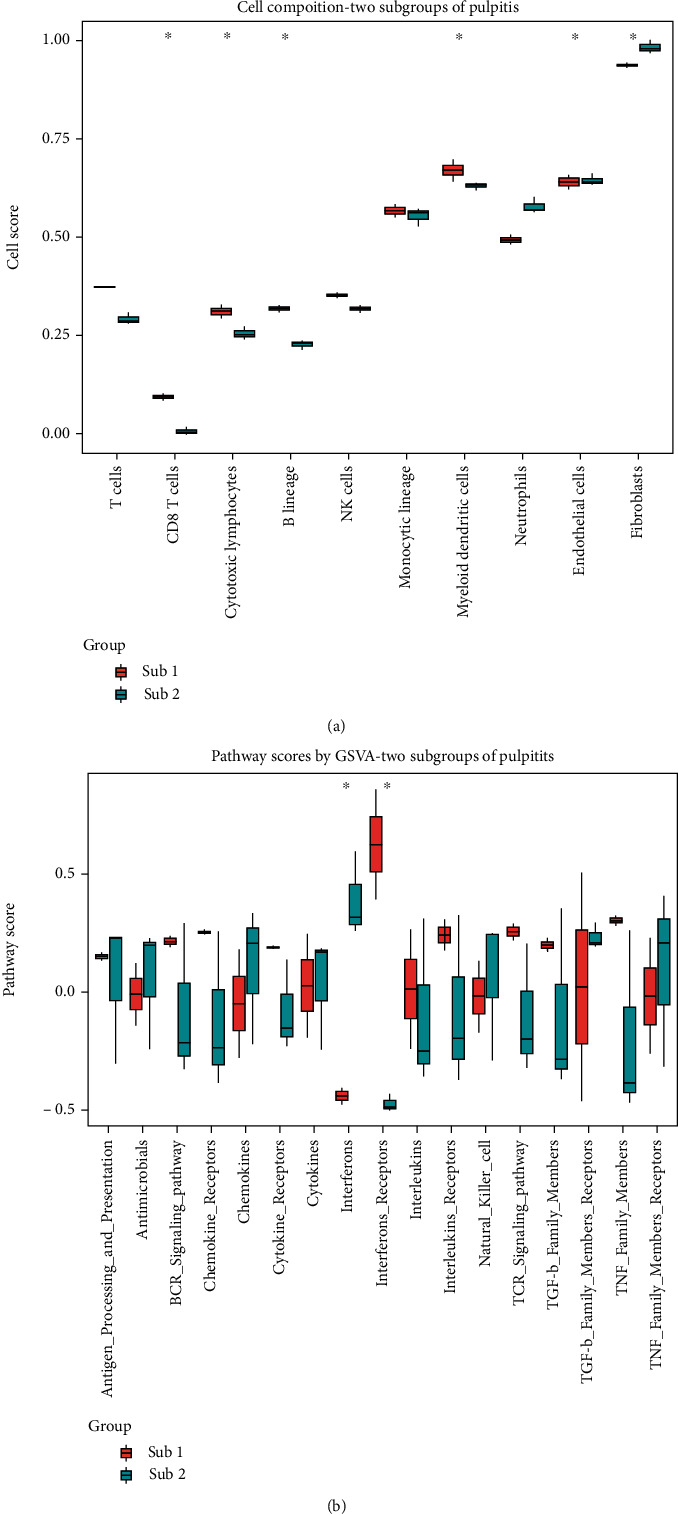
Immune characteristics of pulpitis subgroups. (a) The box plot showed distribution of 10 infiltrating immune cells between two pulpitis immune subgroups. (b) The diversities of 17 immune-related pathways between the two immune subgroups.

To investigate the discrepancy in molecular characterization between the two pulpitis subgroups, we analyzed 17 immune-related pathways and infiltrating immunocytes by gene set variant analysis (GSVA). The expression of infiltrating immunocytes in pulpitis samples was compared between the two subgroups. As shown in Figure 5(a), the levels of CD8 T cells, cytotoxic lymphocytes, B lineage, and myeloid dendritic cells were significantly different between the two groups (*P* < 0.05). Additionally, we analyzed the two subtypes' variations in immune-related pathways. We discovered significant variations in the interferon and interferon receptor pathways between subtypes 1 and 2 (Figure 5(b)). Detailed data information and sample scores are shown in Table S4 and Table S5. From this, we used the heatmap to exhibit the two subtypes' pathway activity of interferons and interferon receptors in the pulpitis samples (Fig. S5).

### 3.6. qRT-PCR Validation of Selected Immune-Related lncRNAs

**Figure 6:**
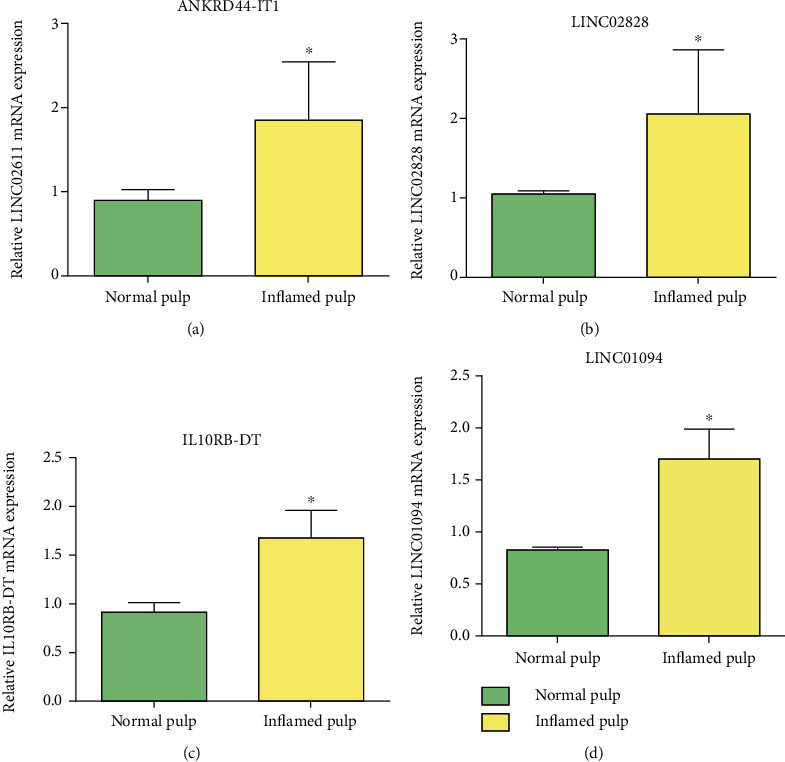
qRT-PCR validation of the selected immune-related lncRNAs. The green columns represented the normal pulp, and yellow columns represented the inflamed pulp (*P* < 0.05).

qRT-PCR was performed to validate the predicted results of immune-related DElncRNAs. We selected the four differentially expressed lncRNA: LINC02828, IL10RB-DT, LINC01094, and ANKRD44-IT1. The results showed that these lncRNAs were upregulated in inflamed pulp tissue (Figure 6).

## 4. Discussion

Pulpitis is an inflammatory condition that affects the pulp tissue. External stimuli, such as bacterial infection or mechanical stimulation, typically initiate the process [24]. Dental pulp stimulates the innate immune system, which may aid in the defense against bacterial invasion. If the infection remains, adaptive immunity is triggered, the dynamic balance between external stimuli and pulp immune defense is broken, and the reversible pulp irritation finally develops into an irreversible inflammatory response [25]. It is vital to understand and accurately analyze the degree of inflammation in dental pulp before initiating therapeutic therapy. According to the most recent AAE guideline [26], patients' clinical symptoms frequently do not accurately reflect the state of their dental pulp at the time of treatment, and the amount of pulp tissue removed or retained is determined by a complex assessment, so identifying key biomarkers is encouraged to improve pulp activity evaluation and clinical decision-making.

Recently, advances in high-throughput RNA detection techniques revealed hitherto undiscovered facets of the human genome. Compared to the well-characterized protein-coding mRNAs, an abundance of noncoding RNA transcripts was discovered. lncRNAs, a type of noncoding RNA, have recently garnered considerable attention due to their critical role in a range of biological processes requiring immune responses [27, 28]. Several studies on the regulation of lncRNAs in pulpitis have been conducted recently. MEG3 lncRNA has been demonstrated as a negative regulator in the pulpitis process [15]. Wang et al. verified the involvement of NUTM2A-AS1 in the initiation and progression of pulpitis [19]. Additionally, an integrated comparative method was used to look for differentially expressed lncRNAs between normal and inflamed pulp tissue [18]. lncRNA expression is highly tissue- and condition-specific [29, 30]. Because various lncRNAs have diverse expression patterns, significantly differently expressed lncRNAs may be employed as ideal disease biomarkers. lncRNAs have been hailed as diagnostic, therapeutic, and prognostic biomarkers in various disciplines of research [31–33]. However, to clarify the formation and progression of pulpitis further, it is critical to investigate the unique regulation mechanism lncRNAs and whether they can be employed as indicators of pulp condition.

To validate the predicted immune-related DElncRNAs, we selected and detected four lncRNAs: LINC02828, IL10RB-DT, LINC01094, and ANKRD44-IT1. Jiang et al. generated an immune-related lncRNA signature in which IL10RB-DT was identified as the immune-related lncRNA with the best prognostic value [34]. LINC01094 was a well-characterized lncRNA involved in proliferation, migration, and epithelial-mesenchymal transition [35]. Nowadays, a study revealed that LINC01094 was an inflammation-related lncRNA that works as a prognostic marker for gastric carcinoma [36]. Additionally, ANKRD44-IT1 has been linked to ulcerative colitis [37]. It is worth noting that this study established the first link between LINC02828 and immune regulation. Our work identified a novel list of lncRNAs associated with the immune response in pulpitis.

Next, we applied a bioinformatic pipeline previously used in pancancer research to discover immune-related lncRNAs in pulpitis [21]. We discovered that 790 lncRNAs were classified into 14 immune-related categories, with the majority being classified as interferons, interferon receptors, and cytokines. The findings indicated that these three pathways might be more important in pulpitis. Additionally, an external independent validation set confirmed the algorithm's reliability for identifying immune-related lncRNAs. We then divided the pulpitis into two subgroups based on particular molecular characteristics. The classification has ever been reported for periodontitis [17], while pulpitis has never been classified in this way. The newly defined subtypes were not based on the classical categorization of pulpitis [38]; rather, they presented a novel classification of pulpitis based on immune-related lncRNAs, which may reflect a finer molecular annotation to different stages of pulpitis. Finally, we investigated 17 immune-related pathways and infiltrating immunocytes in the two groupings to determine why they differed molecularly. The results indicated that, compared to subtype 1, subtype 2 possessed a greater number of active immune gene sets; nevertheless, GSVA analysis revealed that subtype 2 possessed a lower proportion of immunocyte infiltration. The immunosuppression could explain these findings during the strong inflammatory response. When the immunological response is strong or, excessive autoantibodies cause tissue injury, cells in the pulp act as immunosuppressive agents, limiting the maturation and differentiation of immune cells such as CD4+ T lymphocytes dendritic cells [39–41]. Due to the low killing capacity of innate immune cells, the immune system compensates by secreting many cytokines. The results might imply that subtype 2 constituted a late stage of the immunological response that correlated to moderate or severe pulpitis and subtype 1 may reflect initial or mild pulpitis in Wolter's classification [9].

The molecular subtypes of pulpitis in our study may benefit clinical procedures. Currently, the central dilemma of vital pulp therapy is determining the inflammatory status in pulpitis [20]. The most used method clinically is estimating the time of hemostasis at the pulp amputation plane under the surgical microscope. This method only reflects gross blood congestion that may present in some stages of pulpitis or not. Nowadays, chairside portable PCR systems can rapidly detect designated RNA targets in daily clinics, and the differentially expressed genes reported in our study can work as representative candidates of each pulpitis subtype and connect the molecular level evidence to the recently reported finer classification of pulpitis, which, in the end, ease the case selection of vital pulp therapy.

While the integrated algorithm was successfully validated, several limitations remain. First, although we systematically searched the open-source databases and included multiple pulpitis datasets, the total number of pulp samples is still scarce, with each dataset containing only 5-7 samples. As a result, alpha and beta errors may affect predictive accuracy. Second, despite our efforts to clean and reorganize the data to eliminate batch effects and invalid samples, differences persist due to the heterogeneity of the inclusion criterion. Furthermore, although we make the first step for connecting the molecular evidence and clinical classification, more clinical information is still needed to further analyze correlations between molecular findings and clinical characteristics/prognosis for patients, providing a more intuitive explanation for our results. We will collect more detailed patient information in future sample collection, which consist of pulpitis prognosis data, microbiological data, and blood indicator data. Thus, future high-throughput studies involving pulpitis should establish more stringent inclusion criteria, including a larger sample size and the collection of additional clinical data.

In summary, lncRNAs are emerging as critical regulators in immune regulation. Therefore, we systematically identified immune-related lncRNAs in pulpitis and demonstrated the relationship with immune categories. Two immune-related subtypes of pulpitis were distinguished for the first time; the molecular function, cellular component, and relevant pathways were analyzed. The new categorization helps build a finer molecular annotation to distinct stages of pulpitis. We also explored the immune-related lncRNA network which might participate in pulpitis. Our study would ease understanding inflammatory and immune processes in pulpitis at the molecular level.

## Figures and Tables

**Table 1:** The baseline information of each dataset.

GSE accession	GSE77459	GSE92681
Platform	GPL17692	GPL16956
Diagnostic criteria	AAE^1^	AAE
Clinical sample information		
Normal pulp samples	Picked from healthy teeth	Picked from healthy teeth
Inflamed pulp samples	Picked from teeth diagnosed with irreversible pulpitis	Picked from teeth diagnosed with irreversible pulpitis
Number		
Normal pulp samples	6	5
Inflamed pulp samples	6	7

^1^AAE: American Association of Endodontists.

**Table 2:** Details of patients involved in this study.

Number	Age	Sex	Medications	Sample type	Vitality test	Percussion	Spontaneous pain	Diagnosis
01	28Y	M	NAD^1^	Dental pulp	Sensitive to stimulation	-	+	Irreversible pulpitis
02	31Y	F	NAD	Dental pulp	Normal	-	-	Healthy
03	42Y	F	NAD	Dental pulp	Dull to stimulation	-	+	Irreversible pulpitis
04	26Y	F	NAD	Dental pulp	Sensitive to stimulation	+	+	Irreversible pulpitis
05	46Y	F	NAD	Dental pulp	Normal	-	-	Healthy
06	17Y	M	NAD	Dental pulp	Sensitive to stimulation	-	+	Irreversible pulpitis
07	43Y	F	NAD	Dental pulp	Normal	-	-	Healthy
08	32Y	M	NAD	Dental pulp	Dull to stimulation	-	+	Irreversible pulpitis
09	19Y	M	NAD	Dental pulp	Normal	-	-	Healthy
10	21Y	F	NAD	Dental pulp	Sensitive to stimulation	+	+	Irreversible pulpitis
11	25Y	F	NAD	Dental pulp	Normal	-	-	Healthy
12	32Y	M	NAD	Dental pulp	Normal	-	-	Healthy

^1^NAD: no abnormality detected.

## Data Availability

Data can be found in supplementary information files.

## Supplementary Materials

Supplementary 1 Supplementary Figure 1: boxplots for GSE92681. (A) The boxplot of GSE92681. (B) The boxplot of GSE92681 after excluding outliner examples.

Supplementary 2 Supplementary Figure 2: principal component analysis (PCA) results. PCA plots before (A) and after (B) excluding GSM2434473, GSM2434475, and GSM2434484 from GSE92681. Yellow dots: normal samples. Green dots: pulpitis samples.

Supplementary 3 Supplementary Figure 3: volcano plots of differentially expressed mRNAs and lncRNAs inGSE92681. Volcano plots for mRNAs (A) and lncRNAs (B) of GSE92681. Yellow dots: normal samples. Green dots: pulpitis samples (|Log2 fold change (FC)| > 0.5 and *P* < 0.05).

Supplementary 4 Supplementary Figure 4: heatmaps of mRNAs and lncRNAs in the two subgroups. The top 50 DEmRNAs (A) and DElncRNAs (B) with the lowest *P* values in two subgroups were shown by heatmaps.

Supplementary 5 Supplementary Figure 5: heatmap of subgroup 1 and subgroup 2 in the pathways of interferons and interferon receptors.

Supplementary 6 Table S1: the Minimum Information for the Publication of Real-Time Quantitative PCR Experiments (MIQE) guidelines.

Supplementary 7 Table S2: pertinent information on the 38 immune-related DElncRNAs.

Supplementary 8 Table S3: differentially expressed lncRNAs and mRNAs in normal and pulpitis samples.

Supplementary 9 Table S4: infiltrating immunocytes scores between two immune subgroups.

Supplementary 10 Table S5: relative pathway scores between two immune subgroups.
