# Supplementary figures and images for: Identification of Immune-Related lncRNA Regulatory Network in Pulpitis
Source: Dis Markers. 2022 Jun 6;2022:7222092. doi: 10.1155/2022/7222092 (PMC9194960; doi:10.1155/2022/7222092)

**A** Boxplot for GSE92681

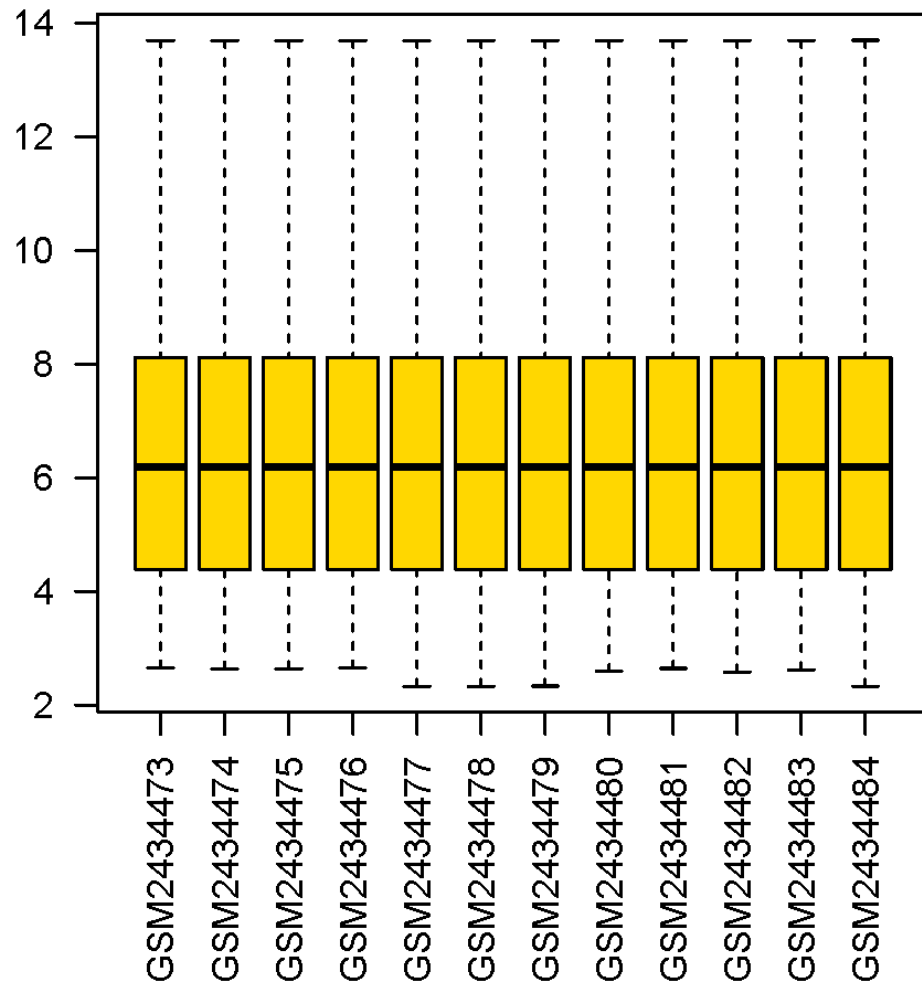

**B** Boxplot for GSE92681 after removing outliers

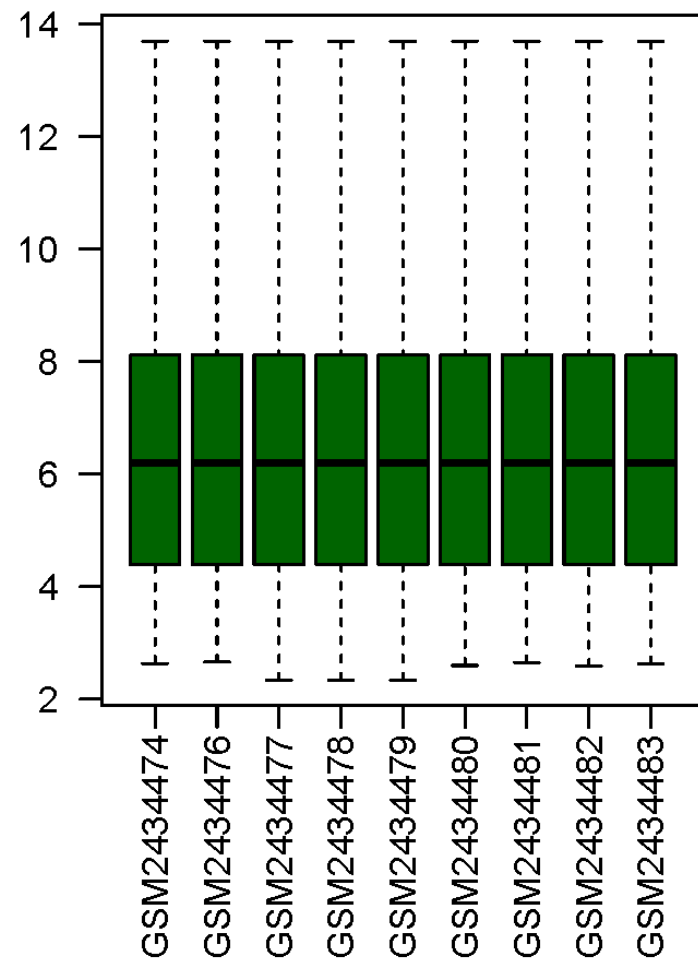

Supplement: Supplementary 1 — Supplementary Figure 1: boxplots for GSE92681. (A) The boxplot of GSE92681. (B) The boxplot of GSE92681 after excluding outliner examples. [file 7222092.f1.pdf]

**A**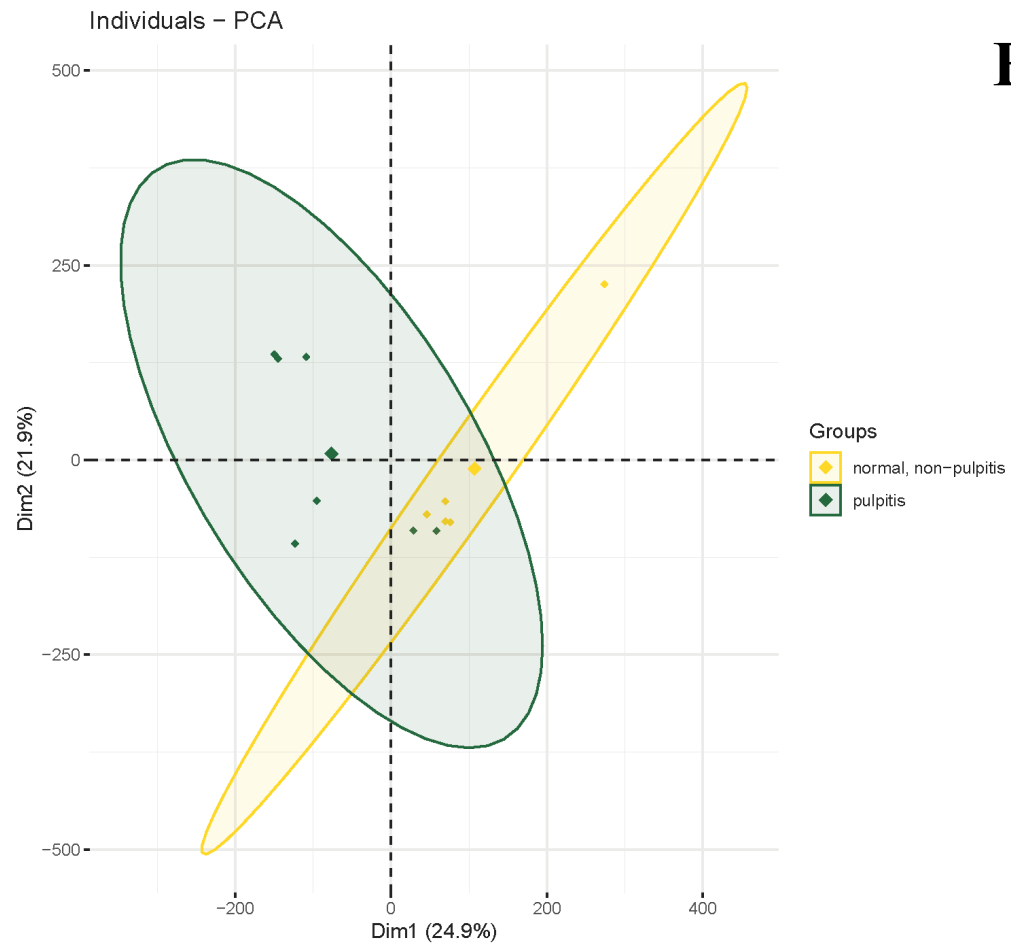**B**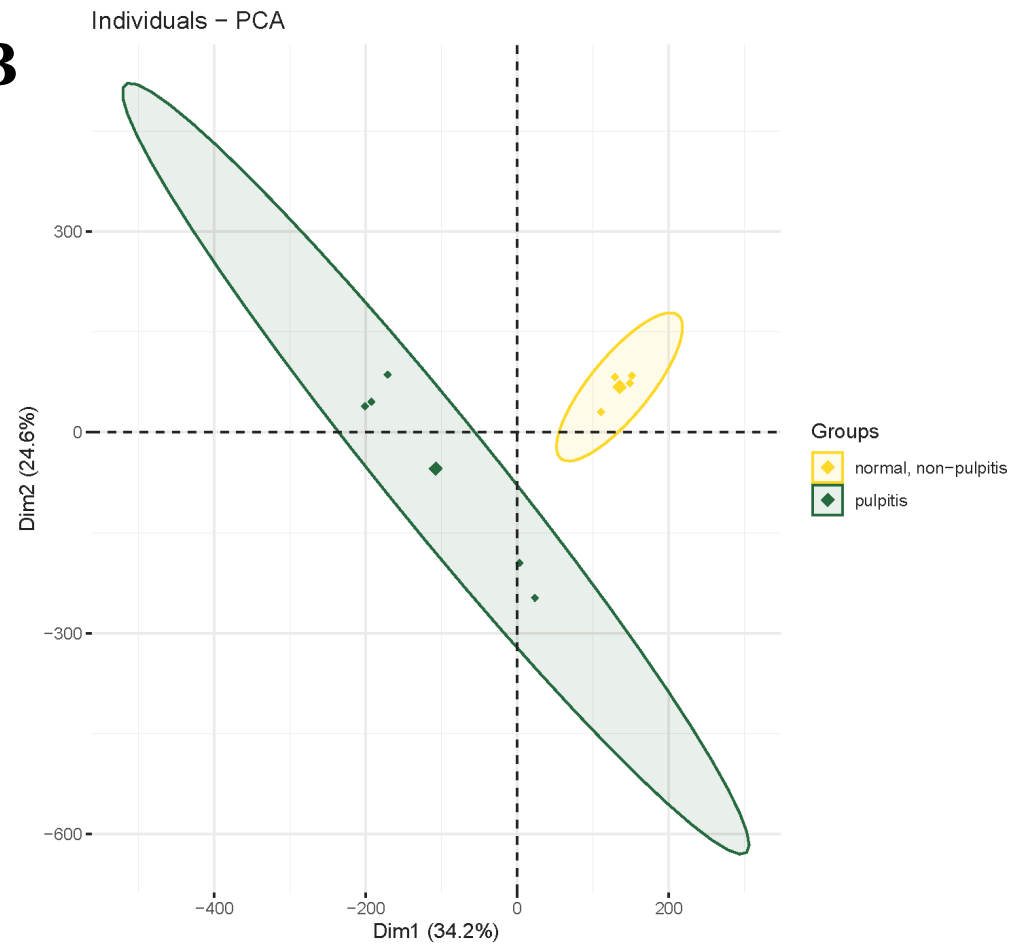

Supplement: Supplementary 2 — Supplementary Figure 2: principal component analysis (PCA) results. PCA plots before (A) and after (B) excluding GSM2434473, GSM2434475, and GSM2434484 from GSE92681. Yellow dots: normal samples. Green dots: pulpitis samples. [file 7222092.f2.pdf]

**A**

Volcano Plot for mRNAs in GSE92681

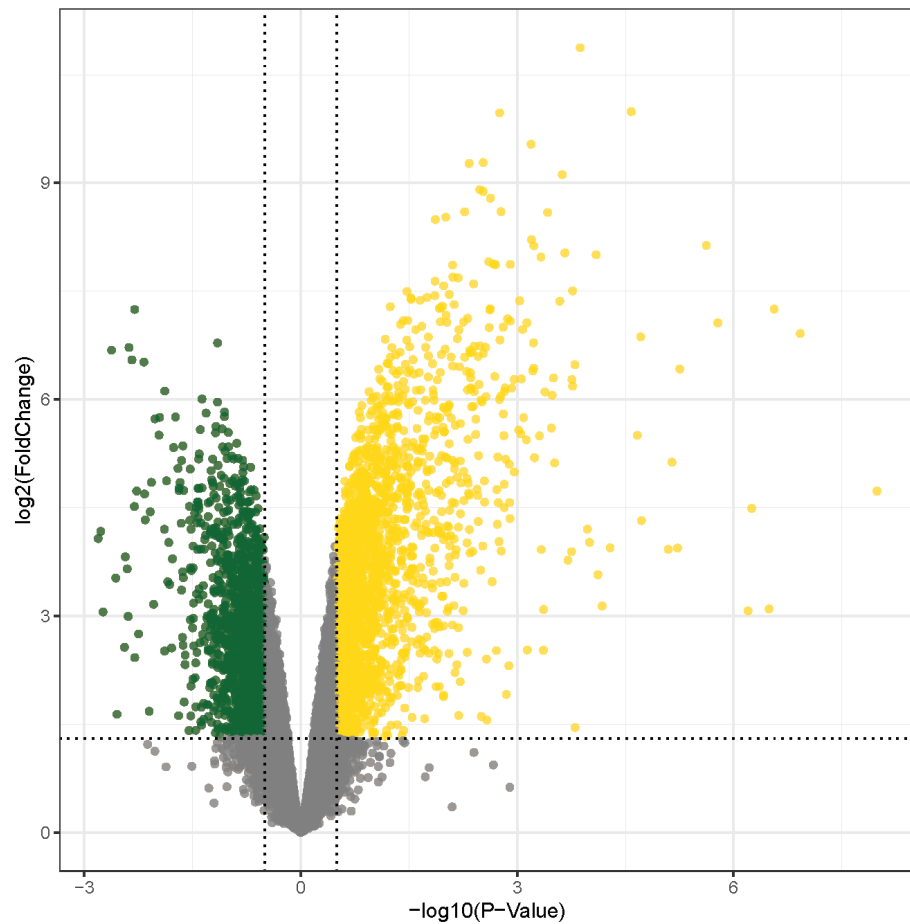**B**

Volcano Plot for ncRNAs in GSE92681

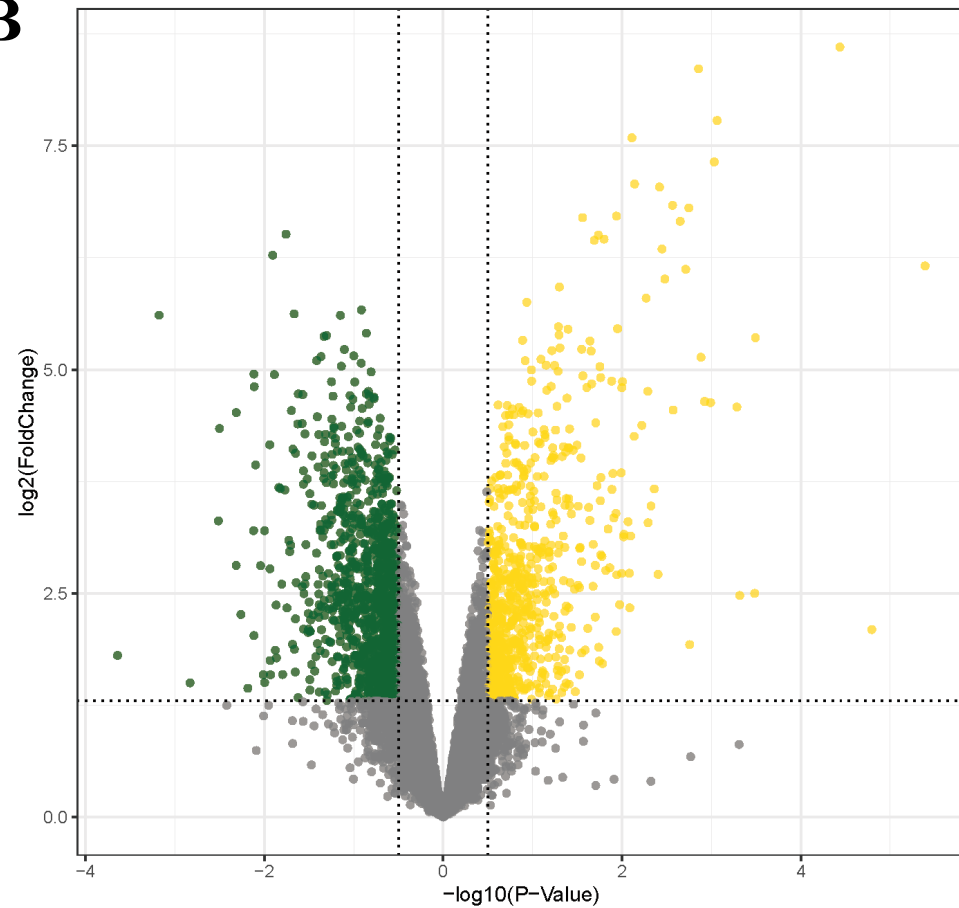

Supplement: Supplementary 3 — Supplementary Figure 3: volcano plots of differentially expressed mRNAs and lncRNAs inGSE92681. Volcano plots for mRNAs (A) and lncRNAs (B) of GSE92681. Yellow dots: normal samples. Green dots: pulpitis samples (|Log2 fold change (FC)| > 0.5 and P < 0.05). [file 7222092.f3.pdf]

**A****Subgroup of pulpitis\_mRNA**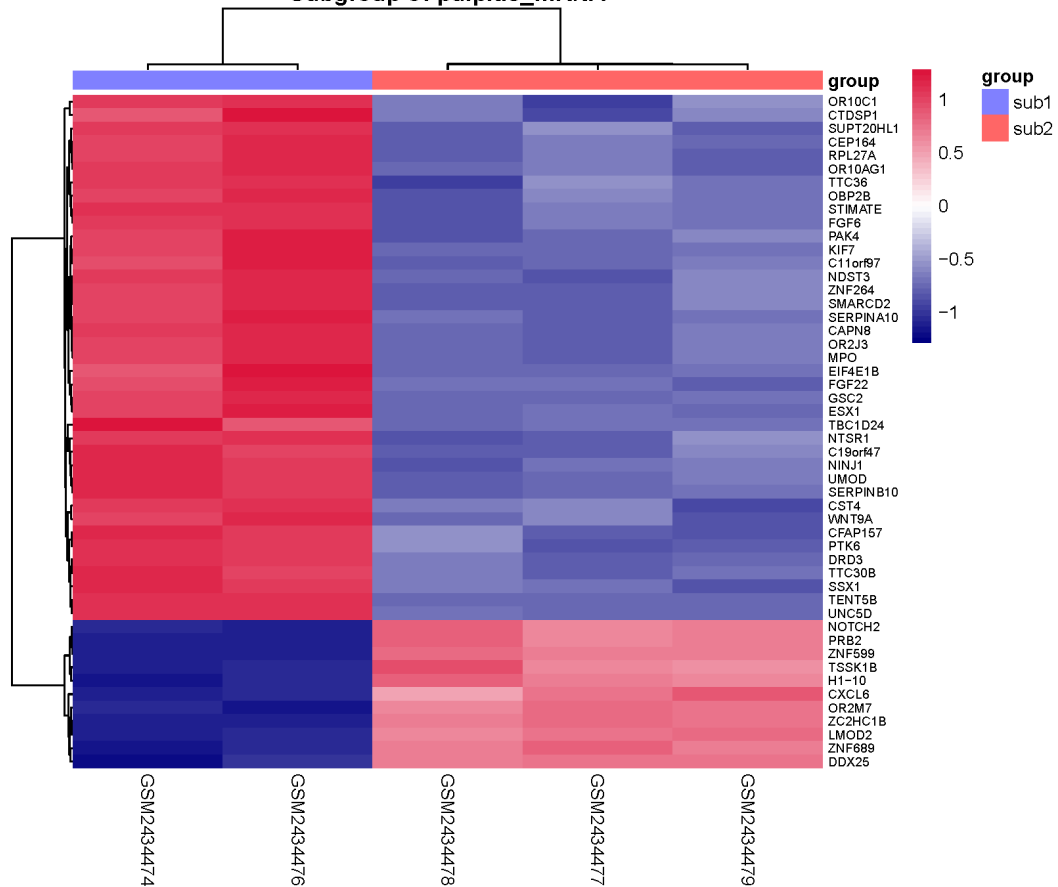**B****Subgroup of pulpitis\_lncRNA**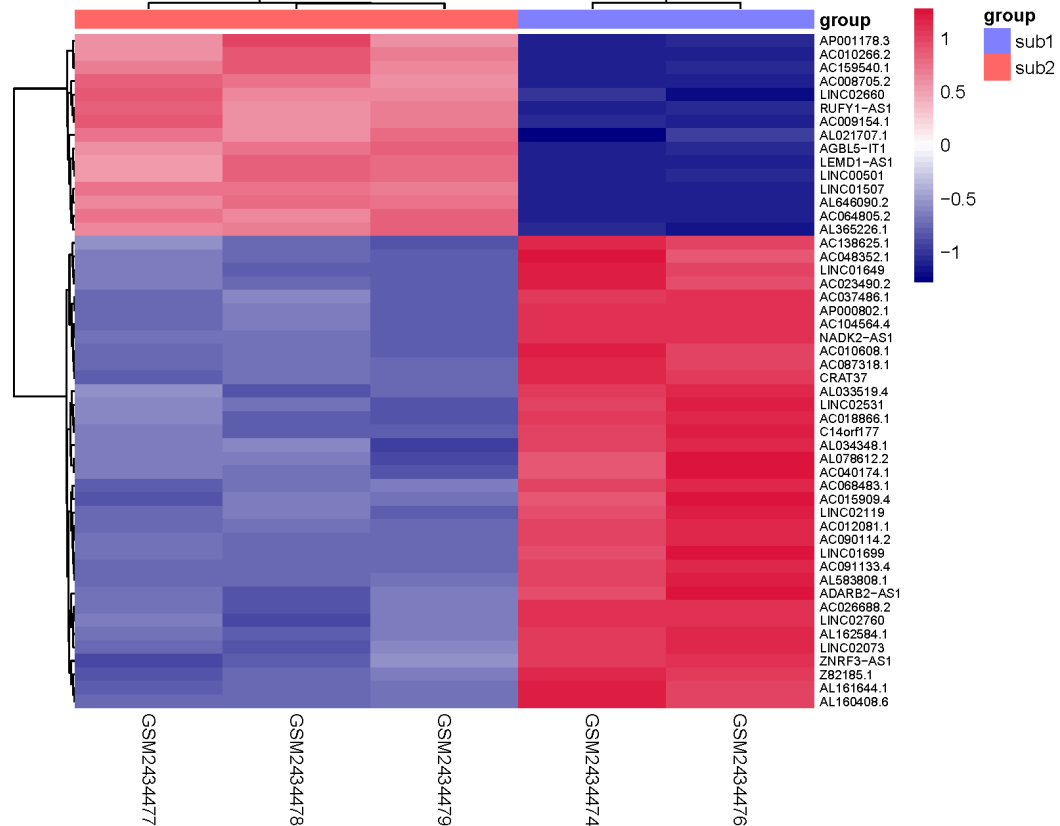

Supplement: Supplementary 4 — Supplementary Figure 4: heatmaps of mRNAs and lncRNAs in the two subgroups. The top 50 DEmRNAs (A) and DElncRNAs (B) with the lowest P values in two subgroups were shown by heatmaps. [file 7222092.f4.pdf]

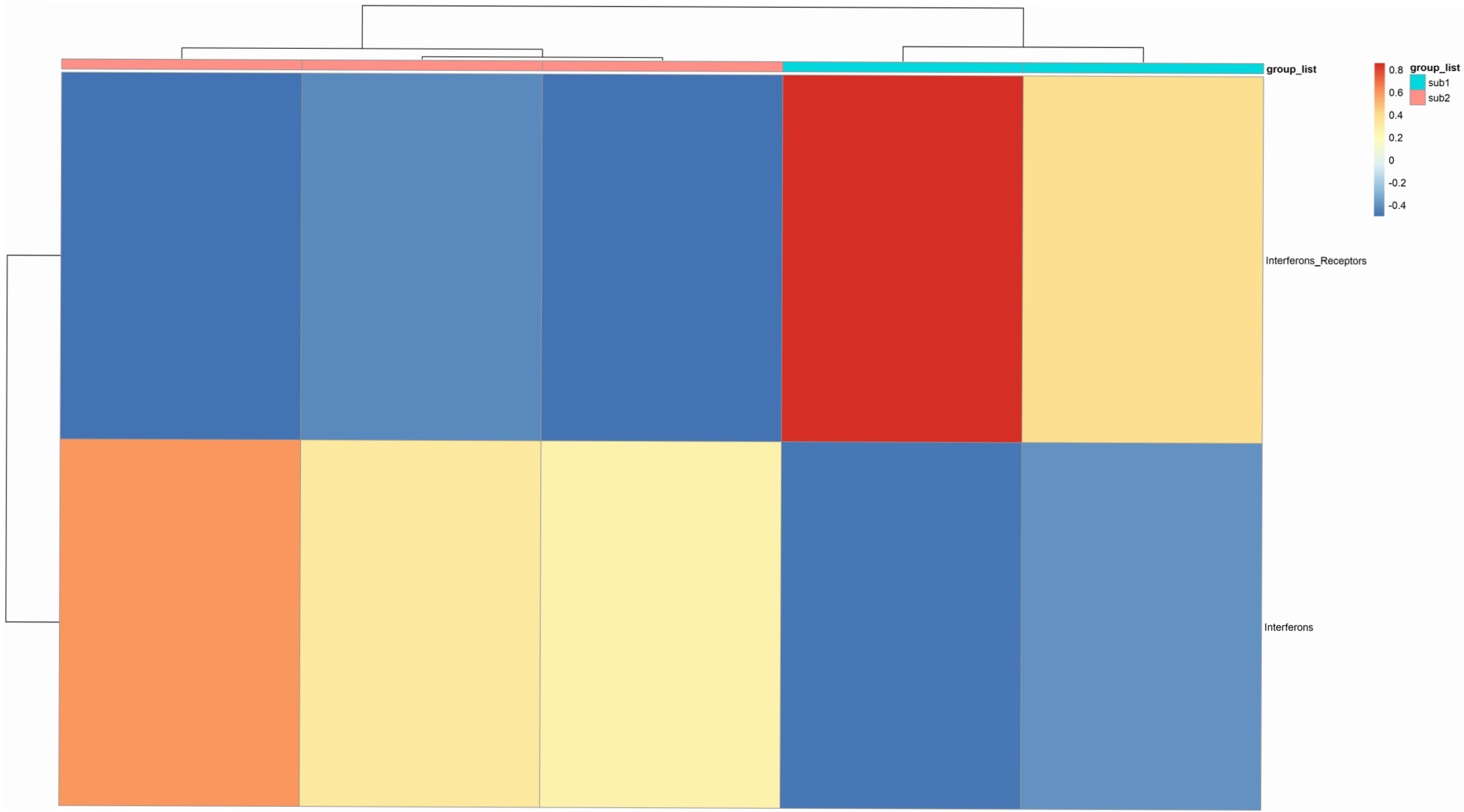

Supplement: Supplementary 5 — Supplementary Figure 5: heatmap of subgroup 1 and subgroup 2 in the pathways of interferons and interferon receptors. [file 7222092.f5.pdf]
