# Supplementary material for: Identification of Immune-Related lncRNA Regulatory Network in Pulpitis
Source: Dis Markers. 2022 Jun 6;2022:7222092. doi: 10.1155/2022/7222092 (PMC9194960; doi:10.1155/2022/7222092)
Supplement: Supplementary 7 — Table S2: pertinent information on the 38 immune-related DElncRNAs. [file 7222092.f7.docx]

| Table S2 Pertinent information on the 38 immune-related DELncRNAs. | | | | | | |
| --- | --- | --- | --- | --- | --- | --- |
| symbol | logFC | AveExpr | t | P.Value | adj.P.Val | B |
| LINC02611 | 1.800107 | 5.536257 | 12.5929 | 3.49E-07 | 2.41E-04 | 7.172457 |
| LINC02828 | 1.690709 | 4.106036 | 12.54952 | 3.60E-07 | 2.41E-04 | 7.14504 |
| IL10RB-DT | 1.562668 | 8.197957 | 8.388965 | 1.17E-05 | 0.002095 | 3.8522 |
| LINC01094 | 1.385779 | 5.69665 | 7.829751 | 2.08E-05 | 0.00271 | 3.285233 |
| AC018607.1 | 2.991986 | 8.556311 | 7.719233 | 2.34E-05 | 0.002912 | 3.16887 |
| AC008074.2 | 1.138236 | 6.816509 | 6.973017 | 5.33E-05 | 0.004308 | 2.343532 |
| AC100778.3 | -1.0832 | 3.341368 | -6.20948 | 1.32E-04 | 0.006652 | 1.422562 |
| ANKRD44-IT1 | 1.093655 | 6.374735 | 5.473547 | 3.42E-04 | 0.011012 | 0.455741 |
| LINC01639 | 1.00273 | 5.419821 | 5.422926 | 3.66E-04 | 0.011553 | 0.386271 |
| AC073257.2 | 1.932367 | 7.417708 | 5.347793 | 4.05E-04 | 0.012345 | 0.282451 |
| LINC00290 | -1.04039 | 3.887172 | -5.30924 | 4.27E-04 | 0.012678 | 0.228848 |
| AC215522.2 | 1.642644 | 5.541109 | 5.216472 | 4.84E-04 | 0.013636 | 0.098943 |
| AC134682.1 | -1.29775 | 6.629086 | -5.15413 | 5.28E-04 | 0.014453 | 0.010922 |
| AC010266.1 | 1.024393 | 4.654822 | 5.094671 | 5.73E-04 | 0.014921 | -0.07359 |
| AC010285.1 | 1.360912 | 6.995309 | 4.723804 | 9.70E-04 | 0.020162 | -0.61262 |
| SH3PXD2A-AS1 | 1.044248 | 7.577101 | 4.689929 | 0.001019 | 0.020687 | -0.66287 |
| PPIC-AS1 | 1.763658 | 10.00806 | 4.593205 | 0.001173 | 0.022062 | -0.80727 |
| LINC01846 | 1.065169 | 4.348858 | 4.348772 | 0.001686 | 0.027355 | -1.17815 |
| AC007540.1 | 1.125894 | 4.128262 | 4.13258 | 0.002339 | 0.033189 | -1.51299 |
| TSBP1-AS1 | -1.07127 | 8.34132 | -4.10786 | 0.002429 | 0.033665 | -1.55167 |
| AC083841.2 | -1.16936 | 3.68891 | -3.94096 | 0.003144 | 0.039109 | -1.81477 |
| AC104777.1 | 1.046318 | 3.668042 | 3.934394 | 0.003176 | 0.039166 | -1.82518 |
| AC046158.1 | -1.19238 | 7.207347 | -3.89203 | 0.003393 | 0.040638 | -1.89252 |
| PDYN-AS1 | -1.13734 | 15.39513 | -3.83934 | 0.003685 | 0.042344 | -1.97657 |
| AC112496.1 | 1.36333 | 8.828743 | 3.643857 | 0.00502 | 0.04968 | -2.2909 |
| AL513324.1 | 1.239348 | 3.408749 | 3.562617 | 0.005716 | 0.05379 | -2.42261 |
| LINC02548 | 1.935688 | 4.213979 | 3.321627 | 0.008438 | 0.067282 | -2.81636 |
| LINC02526 | -1.15612 | 6.261929 | -3.26087 | 0.009318 | 0.071478 | -2.91619 |
| AL355103.1 | -1.08003 | 3.835033 | -3.0807 | 0.012526 | 0.084869 | -3.21315 |
| AC004054.1 | 1.415505 | 6.247505 | 3.030604 | 0.013606 | 0.088833 | -3.29586 |
| GK-AS1 | 1.550053 | 5.528794 | 2.984802 | 0.014677 | 0.092522 | -3.3715 |
| AC009884.1 | -1.32091 | 4.515869 | -2.97757 | 0.014853 | 0.093471 | -3.38344 |
| AL731577.1 | -3.64209 | 7.147032 | -2.94705 | 0.015624 | 0.096374 | -3.43384 |
| KCCAT198 | 1.783255 | 7.960414 | 2.82063 | 0.019275 | 0.108473 | -3.64246 |
| AC015909.2 | 1.525118 | 7.341863 | 2.647462 | 0.025726 | 0.130309 | -3.92721 |
| AC010255.1 | 1.356529 | 4.796686 | 2.457793 | 0.035305 | 0.154475 | -4.23636 |
| DNMT3L-AS1 | -1.20349 | 4.613944 | -2.39665 | 0.039093 | 0.164178 | -4.33511 |
| AC107079.1 | 1.175494 | 4.255062 | 2.326375 | 0.043944 | 0.175196 | -4.4479 |
