# Supplementary material for: Identification of Immune-Related lncRNA Regulatory Network in Pulpitis
Source: Dis Markers. 2022 Jun 6;2022:7222092. doi: 10.1155/2022/7222092 (PMC9194960; doi:10.1155/2022/7222092)
Supplement: Supplementary 8 — Table S3: differentially expressed lncRNAs and mRNAs in normal and pulpitis samples. [file 7222092.f8.docx]

| Table S3 Differentially expressed lncRNAs and mRNAs in normal  and pulpitis samples | |
| --- | --- |
| ID.x | SYMBOL |
| HALLMARK_OXIDATIVE_PHOSPHORYLATION | AC002070.1 |
| HALLMARK_OXIDATIVE_PHOSPHORYLATION | AC002407.1 |
| HALLMARK_INFLAMMATORY_RESPONSE | AC004801.4 |
| HALLMARK_OXIDATIVE_PHOSPHORYLATION | AC004888.1 |
| HALLMARK_INTERFERON_GAMMA_RESPONSE | AC005487.1 |
| HALLMARK_INTERFERON_ALPHA_RESPONSE | AC005487.1 |
| HALLMARK_MYC_TARGETS_V1 | AC005487.1 |
| HALLMARK_OXIDATIVE_PHOSPHORYLATION | AC005487.1 |
| HALLMARK_OXIDATIVE_PHOSPHORYLATION | AC005740.3 |
| HALLMARK_OXIDATIVE_PHOSPHORYLATION | AC006357.1 |
| HALLMARK_OXIDATIVE_PHOSPHORYLATION | AC008074.2 |
| HALLMARK_INTERFERON_GAMMA_RESPONSE | AC008406.2 |
| HALLMARK_INTERFERON_ALPHA_RESPONSE | AC008406.2 |
| HALLMARK_MYC_TARGETS_V1 | AC008406.2 |
| HALLMARK_OXIDATIVE_PHOSPHORYLATION | AC008406.2 |
| HALLMARK_IL6_JAK_STAT3_SIGNALING | AC008406.2 |
| HALLMARK_OXIDATIVE_PHOSPHORYLATION | AC008534.1 |
| HALLMARK_KRAS_SIGNALING_UP | AC008534.1 |
| HALLMARK_MTORC1_SIGNALING | AC008534.1 |
| HALLMARK_INTERFERON_GAMMA_RESPONSE | AC008991.1 |
| HALLMARK_INTERFERON_ALPHA_RESPONSE | AC008991.1 |
| HALLMARK_MYC_TARGETS_V1 | AC008991.1 |
| HALLMARK_OXIDATIVE_PHOSPHORYLATION | AC008991.1 |
| HALLMARK_IL6_JAK_STAT3_SIGNALING | AC008991.1 |
| HALLMARK_OXIDATIVE_PHOSPHORYLATION | AC009908.1 |
| HALLMARK_OXIDATIVE_PHOSPHORYLATION | AC010255.1 |
| HALLMARK_INTERFERON_GAMMA_RESPONSE | AC010285.1 |
| HALLMARK_INTERFERON_ALPHA_RESPONSE | AC010285.1 |
| HALLMARK_MYC_TARGETS_V1 | AC010285.1 |
| HALLMARK_OXIDATIVE_PHOSPHORYLATION | AC010285.1 |
| HALLMARK_IL6_JAK_STAT3_SIGNALING | AC010285.1 |
| HALLMARK_OXIDATIVE_PHOSPHORYLATION | AC010468.1 |
| HALLMARK_OXIDATIVE_PHOSPHORYLATION | AC011352.3 |
| HALLMARK_OXIDATIVE_PHOSPHORYLATION | AC011603.2 |
| HALLMARK_INTERFERON_GAMMA_RESPONSE | AC012050.1 |
| HALLMARK_INTERFERON_ALPHA_RESPONSE | AC012050.1 |
| HALLMARK_MYC_TARGETS_V1 | AC012050.1 |
| HALLMARK_OXIDATIVE_PHOSPHORYLATION | AC012050.1 |
| HALLMARK_IL6_JAK_STAT3_SIGNALING | AC012050.1 |
| HALLMARK_OXIDATIVE_PHOSPHORYLATION | AC012404.1 |
| HALLMARK_INFLAMMATORY_RESPONSE | AC012464.2 |
| HALLMARK_INTERFERON_GAMMA_RESPONSE | AC012464.2 |
| HALLMARK_INFLAMMATORY_RESPONSE | AC015631.1 |
| HALLMARK_OXIDATIVE_PHOSPHORYLATION | AC018607.1 |
| HALLMARK_TNFA_SIGNALING_VIA_NFKB | AC020892.2 |
| HALLMARK_APOPTOSIS | AC020892.2 |
| HALLMARK_INTERFERON_GAMMA_RESPONSE | AC020892.2 |
| HALLMARK_MYC_TARGETS_V1 | AC020892.2 |
| HALLMARK_INFLAMMATORY_RESPONSE | AC020892.2 |
| HALLMARK_IL2_STAT5_SIGNALING | AC020892.2 |
| HALLMARK_TNFA_SIGNALING_VIA_NFKB | AC022400.1 |
| HALLMARK_APOPTOSIS | AC022400.1 |
| HALLMARK_INTERFERON_GAMMA_RESPONSE | AC022400.1 |
| HALLMARK_MYC_TARGETS_V1 | AC022400.1 |
| HALLMARK_INFLAMMATORY_RESPONSE | AC022400.1 |
| HALLMARK_IL2_STAT5_SIGNALING | AC022400.1 |
| HALLMARK_TNFA_SIGNALING_VIA_NFKB | AC022816.1 |
| HALLMARK_INTERFERON_GAMMA_RESPONSE | AC022816.1 |
| HALLMARK_APOPTOSIS | AC022816.1 |
| HALLMARK_INFLAMMATORY_RESPONSE | AC022816.1 |
| HALLMARK_MYC_TARGETS_V1 | AC022816.1 |
| HALLMARK_IL2_STAT5_SIGNALING | AC022816.1 |
| HALLMARK_INFLAMMATORY_RESPONSE | AC023824.5 |
| HALLMARK_INFLAMMATORY_RESPONSE | AC023824.5 |
| HALLMARK_INTERFERON_GAMMA_RESPONSE | AC023824.5 |
| HALLMARK_INTERFERON_GAMMA_RESPONSE | AC023824.5 |
| HALLMARK_OXIDATIVE_PHOSPHORYLATION | AC025437.2 |
| HALLMARK_OXIDATIVE_PHOSPHORYLATION | AC026979.1 |
| HALLMARK_TNFA_SIGNALING_VIA_NFKB | AC032019.1 |
| HALLMARK_APOPTOSIS | AC032019.1 |
| HALLMARK_INTERFERON_GAMMA_RESPONSE | AC032019.1 |
| HALLMARK_MYC_TARGETS_V1 | AC032019.1 |
| HALLMARK_INFLAMMATORY_RESPONSE | AC032019.1 |
| HALLMARK_IL2_STAT5_SIGNALING | AC032019.1 |
| HALLMARK_INTERFERON_GAMMA_RESPONSE | AC034102.3 |
| HALLMARK_INTERFERON_ALPHA_RESPONSE | AC034102.3 |
| HALLMARK_MYC_TARGETS_V1 | AC034102.3 |
| HALLMARK_OXIDATIVE_PHOSPHORYLATION | AC034102.3 |
| HALLMARK_IL6_JAK_STAT3_SIGNALING | AC034102.3 |
| HALLMARK_INFLAMMATORY_RESPONSE | AC036101.1 |
| HALLMARK_KRAS_SIGNALING_UP | AC036101.1 |
| HALLMARK_TNFA_SIGNALING_VIA_NFKB | AC036101.1 |
| HALLMARK_SPERMATOGENESIS | AC036101.1 |
| HALLMARK_INTERFERON_GAMMA_RESPONSE | AC046158.1 |
| HALLMARK_INTERFERON_ALPHA_RESPONSE | AC046158.1 |
| HALLMARK_MYC_TARGETS_V1 | AC046158.1 |
| HALLMARK_OXIDATIVE_PHOSPHORYLATION | AC046158.1 |
| HALLMARK_IL6_JAK_STAT3_SIGNALING | AC046158.1 |
| HALLMARK_INFLAMMATORY_RESPONSE | AC069410.1 |
| HALLMARK_KRAS_SIGNALING_UP | AC069410.1 |
| HALLMARK_TNFA_SIGNALING_VIA_NFKB | AC069410.1 |
| HALLMARK_SPERMATOGENESIS | AC069410.1 |
| HALLMARK_TNFA_SIGNALING_VIA_NFKB | AC073115.1 |
| HALLMARK_ALLOGRAFT_REJECTION | AC073115.1 |
| HALLMARK_INFLAMMATORY_RESPONSE | AC073115.1 |
| HALLMARK_PI3K_AKT_MTOR_SIGNALING | AC073115.1 |
| HALLMARK_OXIDATIVE_PHOSPHORYLATION | AC073188.3 |
| HALLMARK_OXIDATIVE_PHOSPHORYLATION | AC073257.2 |
| HALLMARK_OXIDATIVE_PHOSPHORYLATION | AC078865.1 |
| HALLMARK_OXIDATIVE_PHOSPHORYLATION | AC079070.1 |
| HALLMARK_OXIDATIVE_PHOSPHORYLATION | AC079866.2 |
| HALLMARK_EPITHELIAL_MESENCHYMAL_TRANSITION | AC079866.2 |
| HALLMARK_MYC_TARGETS_V1 | AC079866.2 |
| HALLMARK_INTERFERON_ALPHA_RESPONSE | AC079866.2 |
| HALLMARK_DNA_REPAIR | AC079866.2 |
| HALLMARK_INTERFERON_GAMMA_RESPONSE | AC079866.2 |
| HALLMARK_COMPLEMENT | AC079866.2 |
| HALLMARK_INTERFERON_GAMMA_RESPONSE | AC080023.1 |
| HALLMARK_INTERFERON_ALPHA_RESPONSE | AC080023.1 |
| HALLMARK_MYC_TARGETS_V1 | AC080023.1 |
| HALLMARK_OXIDATIVE_PHOSPHORYLATION | AC080023.1 |
| HALLMARK_INFLAMMATORY_RESPONSE | AC080037.2 |
| HALLMARK_INFLAMMATORY_RESPONSE | AC080037.2 |
| HALLMARK_TNFA_SIGNALING_VIA_NFKB | AC080037.2 |
| HALLMARK_TNFA_SIGNALING_VIA_NFKB | AC080037.2 |
| HALLMARK_SPERMATOGENESIS | AC080037.2 |
| HALLMARK_SPERMATOGENESIS | AC080037.2 |
| HALLMARK_KRAS_SIGNALING_UP | AC080037.2 |
| HALLMARK_KRAS_SIGNALING_UP | AC080037.2 |
| HALLMARK_INTERFERON_GAMMA_RESPONSE | AC090517.5 |
| HALLMARK_INTERFERON_ALPHA_RESPONSE | AC090517.5 |
| HALLMARK_MYC_TARGETS_V1 | AC090517.5 |
| HALLMARK_OXIDATIVE_PHOSPHORYLATION | AC090517.5 |
| HALLMARK_INTERFERON_GAMMA_RESPONSE | AC092756.1 |
| HALLMARK_INTERFERON_ALPHA_RESPONSE | AC092756.1 |
| HALLMARK_MYC_TARGETS_V1 | AC092756.1 |
| HALLMARK_OXIDATIVE_PHOSPHORYLATION | AC092756.1 |
| HALLMARK_IL6_JAK_STAT3_SIGNALING | AC092756.1 |
| HALLMARK_INTERFERON_GAMMA_RESPONSE | AC093157.1 |
| HALLMARK_INTERFERON_ALPHA_RESPONSE | AC093157.1 |
| HALLMARK_MYC_TARGETS_V1 | AC093157.1 |
| HALLMARK_OXIDATIVE_PHOSPHORYLATION | AC093157.1 |
| HALLMARK_INTERFERON_GAMMA_RESPONSE | AC093673.1 |
| HALLMARK_INTERFERON_ALPHA_RESPONSE | AC093673.1 |
| HALLMARK_MYC_TARGETS_V1 | AC093673.1 |
| HALLMARK_OXIDATIVE_PHOSPHORYLATION | AC093673.1 |
| HALLMARK_IL6_JAK_STAT3_SIGNALING | AC093673.1 |
| HALLMARK_OXIDATIVE_PHOSPHORYLATION | AC096577.1 |
| HALLMARK_INTERFERON_GAMMA_RESPONSE | AC099509.2 |
| HALLMARK_INTERFERON_ALPHA_RESPONSE | AC099509.2 |
| HALLMARK_MYC_TARGETS_V1 | AC099509.2 |
| HALLMARK_OXIDATIVE_PHOSPHORYLATION | AC099509.2 |
| HALLMARK_INTERFERON_GAMMA_RESPONSE | AC104316.2 |
| HALLMARK_INTERFERON_ALPHA_RESPONSE | AC104316.2 |
| HALLMARK_MYC_TARGETS_V1 | AC104316.2 |
| HALLMARK_OXIDATIVE_PHOSPHORYLATION | AC104316.2 |
| HALLMARK_IL6_JAK_STAT3_SIGNALING | AC104316.2 |
| HALLMARK_INFLAMMATORY_RESPONSE | AC106744.2 |
| HALLMARK_INFLAMMATORY_RESPONSE | AC106744.2 |
| HALLMARK_OXIDATIVE_PHOSPHORYLATION | AC106864.1 |
| HALLMARK_OXIDATIVE_PHOSPHORYLATION | AC107208.1 |
| HALLMARK_TNFA_SIGNALING_VIA_NFKB | AC109479.3 |
| HALLMARK_APOPTOSIS | AC109479.3 |
| HALLMARK_INTERFERON_GAMMA_RESPONSE | AC109479.3 |
| HALLMARK_INFLAMMATORY_RESPONSE | AC109479.3 |
| HALLMARK_MYC_TARGETS_V1 | AC109479.3 |
| HALLMARK_IL2_STAT5_SIGNALING | AC109479.3 |
| HALLMARK_INTERFERON_GAMMA_RESPONSE | AC112496.1 |
| HALLMARK_INTERFERON_ALPHA_RESPONSE | AC112496.1 |
| HALLMARK_MYC_TARGETS_V1 | AC112496.1 |
| HALLMARK_OXIDATIVE_PHOSPHORYLATION | AC112496.1 |
| HALLMARK_OXIDATIVE_PHOSPHORYLATION | AC113143.1 |
| HALLMARK_INFLAMMATORY_RESPONSE | AC113414.1 |
| HALLMARK_INFLAMMATORY_RESPONSE | AC113414.1 |
| HALLMARK_MTORC1_SIGNALING | AC114814.1 |
| HALLMARK_OXIDATIVE_PHOSPHORYLATION | AC114814.1 |
| HALLMARK_INTERFERON_GAMMA_RESPONSE | AC116096.1 |
| HALLMARK_INTERFERON_ALPHA_RESPONSE | AC116096.1 |
| HALLMARK_MYC_TARGETS_V1 | AC116096.1 |
| HALLMARK_OXIDATIVE_PHOSPHORYLATION | AC116096.1 |
| HALLMARK_INFLAMMATORY_RESPONSE | AC116362.1 |
| HALLMARK_INFLAMMATORY_RESPONSE | AC116362.1 |
| HALLMARK_INTERFERON_GAMMA_RESPONSE | AC116362.1 |
| HALLMARK_INTERFERON_GAMMA_RESPONSE | AC116362.1 |
| HALLMARK_TNFA_SIGNALING_VIA_NFKB | AC124798.1 |
| HALLMARK_APOPTOSIS | AC124798.1 |
| HALLMARK_INTERFERON_GAMMA_RESPONSE | AC124798.1 |
| HALLMARK_INFLAMMATORY_RESPONSE | AC124798.1 |
| HALLMARK_MYC_TARGETS_V1 | AC124798.1 |
| HALLMARK_IL2_STAT5_SIGNALING | AC124798.1 |
| HALLMARK_OXIDATIVE_PHOSPHORYLATION | AC127894.1 |
| HALLMARK_INTERFERON_GAMMA_RESPONSE | AC129926.1 |
| HALLMARK_INTERFERON_ALPHA_RESPONSE | AC129926.1 |
| HALLMARK_MYC_TARGETS_V1 | AC129926.1 |
| HALLMARK_OXIDATIVE_PHOSPHORYLATION | AC129926.1 |
| HALLMARK_IL6_JAK_STAT3_SIGNALING | AC129926.1 |
| HALLMARK_TNFA_SIGNALING_VIA_NFKB | AC132938.2 |
| HALLMARK_HYPOXIA | AC132938.2 |
| HALLMARK_UNFOLDED_PROTEIN_RESPONSE | AC132938.2 |
| HALLMARK_MYC_TARGETS_V1 | AC132938.2 |
| HALLMARK_INTERFERON_GAMMA_RESPONSE | AC136475.5 |
| HALLMARK_INTERFERON_ALPHA_RESPONSE | AC136475.5 |
| HALLMARK_MYC_TARGETS_V1 | AC136475.5 |
| HALLMARK_OXIDATIVE_PHOSPHORYLATION | AC136475.5 |
| HALLMARK_IL6_JAK_STAT3_SIGNALING | AC136475.5 |
| HALLMARK_OXIDATIVE_PHOSPHORYLATION | AC138331.1 |
| HALLMARK_OXIDATIVE_PHOSPHORYLATION | AC138915.3 |
| HALLMARK_INTERFERON_GAMMA_RESPONSE | AC215522.2 |
| HALLMARK_INTERFERON_ALPHA_RESPONSE | AC215522.2 |
| HALLMARK_MYC_TARGETS_V1 | AC215522.2 |
| HALLMARK_OXIDATIVE_PHOSPHORYLATION | AC215522.2 |
| HALLMARK_IL6_JAK_STAT3_SIGNALING | AC215522.2 |
| HALLMARK_INTERFERON_GAMMA_RESPONSE | AC245297.3 |
| HALLMARK_INTERFERON_ALPHA_RESPONSE | AC245297.3 |
| HALLMARK_MYC_TARGETS_V1 | AC245297.3 |
| HALLMARK_OXIDATIVE_PHOSPHORYLATION | AC245297.3 |
| HALLMARK_IL6_JAK_STAT3_SIGNALING | AC245297.3 |
| HALLMARK_OXIDATIVE_PHOSPHORYLATION | AF235103.1 |
| HALLMARK_OXIDATIVE_PHOSPHORYLATION | AL021707.1 |
| HALLMARK_OXIDATIVE_PHOSPHORYLATION | AL049836.2 |
| HALLMARK_INFLAMMATORY_RESPONSE | AL117190.2 |
| HALLMARK_OXIDATIVE_PHOSPHORYLATION | AL136140.1 |
| HALLMARK_INTERFERON_GAMMA_RESPONSE | AL137003.1 |
| HALLMARK_INTERFERON_ALPHA_RESPONSE | AL137003.1 |
| HALLMARK_MYC_TARGETS_V1 | AL137003.1 |
| HALLMARK_OXIDATIVE_PHOSPHORYLATION | AL137003.1 |
| HALLMARK_OXIDATIVE_PHOSPHORYLATION | AL138690.1 |
| HALLMARK_INTERFERON_GAMMA_RESPONSE | AL157400.2 |
| HALLMARK_INTERFERON_ALPHA_RESPONSE | AL157400.2 |
| HALLMARK_MYC_TARGETS_V1 | AL157400.2 |
| HALLMARK_OXIDATIVE_PHOSPHORYLATION | AL157400.2 |
| HALLMARK_IL6_JAK_STAT3_SIGNALING | AL157400.2 |
| HALLMARK_MYC_TARGETS_V1 | AL158166.1 |
| HALLMARK_TNFA_SIGNALING_VIA_NFKB | AL158166.1 |
| HALLMARK_INFLAMMATORY_RESPONSE | AL162595.1 |
| HALLMARK_INTERFERON_GAMMA_RESPONSE | AL162595.1 |
| HALLMARK_INTERFERON_GAMMA_RESPONSE | AL162595.2 |
| HALLMARK_INTERFERON_ALPHA_RESPONSE | AL162595.2 |
| HALLMARK_MYC_TARGETS_V1 | AL162595.2 |
| HALLMARK_OXIDATIVE_PHOSPHORYLATION | AL162595.2 |
| HALLMARK_OXIDATIVE_PHOSPHORYLATION | AL353680.1 |
| HALLMARK_MTORC1_SIGNALING | AL353680.1 |
| HALLMARK_INTERFERON_GAMMA_RESPONSE | AL354936.1 |
| HALLMARK_INTERFERON_ALPHA_RESPONSE | AL354936.1 |
| HALLMARK_MYC_TARGETS_V1 | AL354936.1 |
| HALLMARK_OXIDATIVE_PHOSPHORYLATION | AL354936.1 |
| HALLMARK_OXIDATIVE_PHOSPHORYLATION | AL358334.2 |
| HALLMARK_OXIDATIVE_PHOSPHORYLATION | AL360268.1 |
| HALLMARK_OXIDATIVE_PHOSPHORYLATION | AL390067.1 |
| HALLMARK_INFLAMMATORY_RESPONSE | AL391557.1 |
| HALLMARK_INFLAMMATORY_RESPONSE | AL391557.1 |
| HALLMARK_INFLAMMATORY_RESPONSE | AL391557.1 |
| HALLMARK_INTERFERON_GAMMA_RESPONSE | AL450992.1 |
| HALLMARK_INTERFERON_ALPHA_RESPONSE | AL450992.1 |
| HALLMARK_MYC_TARGETS_V1 | AL450992.1 |
| HALLMARK_OXIDATIVE_PHOSPHORYLATION | AL450992.1 |
| HALLMARK_OXIDATIVE_PHOSPHORYLATION | AL627309.1 |
| HALLMARK_INFLAMMATORY_RESPONSE | AL645937.2 |
| HALLMARK_INFLAMMATORY_RESPONSE | AL772337.2 |
| HALLMARK_INFLAMMATORY_RESPONSE | AL772337.2 |
| HALLMARK_INFLAMMATORY_RESPONSE | AL772337.2 |
| HALLMARK_OXIDATIVE_PHOSPHORYLATION | AP000547.3 |
| HALLMARK_INTERFERON_GAMMA_RESPONSE | AP001011.1 |
| HALLMARK_INTERFERON_ALPHA_RESPONSE | AP001011.1 |
| HALLMARK_MYC_TARGETS_V1 | AP001011.1 |
| HALLMARK_OXIDATIVE_PHOSPHORYLATION | AP001011.1 |
| HALLMARK_IL6_JAK_STAT3_SIGNALING | AP001011.1 |
| HALLMARK_OXIDATIVE_PHOSPHORYLATION | AP001453.2 |
| HALLMARK_MYC_TARGETS_V1 | AP002856.1 |
| HALLMARK_TNFA_SIGNALING_VIA_NFKB | AP002856.1 |
| HALLMARK_OXIDATIVE_PHOSPHORYLATION | AP003066.1 |
| HALLMARK_INFLAMMATORY_RESPONSE | AP003550.1 |
| HALLMARK_TNFA_SIGNALING_VIA_NFKB | AP003550.1 |
| HALLMARK_KRAS_SIGNALING_UP | AP003550.1 |
| HALLMARK_ALLOGRAFT_REJECTION | AP003550.1 |
| HALLMARK_SPERMATOGENESIS | AP003550.1 |
| HALLMARK_OXIDATIVE_PHOSPHORYLATION | AP004550.1 |
| HALLMARK_INTERFERON_GAMMA_RESPONSE | AP005202.2 |
| HALLMARK_INTERFERON_ALPHA_RESPONSE | AP005202.2 |
| HALLMARK_MYC_TARGETS_V1 | AP005202.2 |
| HALLMARK_OXIDATIVE_PHOSPHORYLATION | AP005202.2 |
| HALLMARK_INTERFERON_GAMMA_RESPONSE | ARAP1-AS2 |
| HALLMARK_INTERFERON_ALPHA_RESPONSE | ARAP1-AS2 |
| HALLMARK_MYC_TARGETS_V1 | ARAP1-AS2 |
| HALLMARK_OXIDATIVE_PHOSPHORYLATION | ARAP1-AS2 |
| HALLMARK_OXIDATIVE_PHOSPHORYLATION | C1QTNF9-AS1 |
| HALLMARK_INTERFERON_GAMMA_RESPONSE | DAAM2-AS1 |
| HALLMARK_INTERFERON_ALPHA_RESPONSE | DAAM2-AS1 |
| HALLMARK_MYC_TARGETS_V1 | DAAM2-AS1 |
| HALLMARK_OXIDATIVE_PHOSPHORYLATION | DAAM2-AS1 |
| HALLMARK_IL6_JAK_STAT3_SIGNALING | DAAM2-AS1 |
| HALLMARK_INTERFERON_GAMMA_RESPONSE | DANCR |
| HALLMARK_INTERFERON_ALPHA_RESPONSE | DANCR |
| HALLMARK_MYC_TARGETS_V1 | DANCR |
| HALLMARK_OXIDATIVE_PHOSPHORYLATION | DANCR |
| HALLMARK_OXIDATIVE_PHOSPHORYLATION | EHHADH-AS1 |
| HALLMARK_MYC_TARGETS_V1 | FAM225A |
| HALLMARK_TNFA_SIGNALING_VIA_NFKB | FAM225A |
| HALLMARK_INTERFERON_GAMMA_RESPONSE | GK-AS1 |
| HALLMARK_INTERFERON_ALPHA_RESPONSE | GK-AS1 |
| HALLMARK_MYC_TARGETS_V1 | GK-AS1 |
| HALLMARK_OXIDATIVE_PHOSPHORYLATION | GK-AS1 |
| HALLMARK_TNFA_SIGNALING_VIA_NFKB | GNAS-AS1 |
| HALLMARK_APOPTOSIS | GNAS-AS1 |
| HALLMARK_INTERFERON_GAMMA_RESPONSE | GNAS-AS1 |
| HALLMARK_INFLAMMATORY_RESPONSE | GNAS-AS1 |
| HALLMARK_IL2_STAT5_SIGNALING | GNAS-AS1 |
| HALLMARK_MYC_TARGETS_V1 | GNAS-AS1 |
| HALLMARK_OXIDATIVE_PHOSPHORYLATION | IGBP1-AS1 |
| HALLMARK_INFLAMMATORY_RESPONSE | IL10RB-DT |
| HALLMARK_TNFA_SIGNALING_VIA_NFKB | ITGB2-AS1 |
| HALLMARK_APOPTOSIS | ITGB2-AS1 |
| HALLMARK_INTERFERON_GAMMA_RESPONSE | ITGB2-AS1 |
| HALLMARK_INFLAMMATORY_RESPONSE | ITGB2-AS1 |
| HALLMARK_MYC_TARGETS_V1 | ITGB2-AS1 |
| HALLMARK_IL2_STAT5_SIGNALING | ITGB2-AS1 |
| HALLMARK_MTORC1_SIGNALING | KCNQ2-AS1 |
| HALLMARK_OXIDATIVE_PHOSPHORYLATION | KCNQ2-AS1 |
| HALLMARK_OXIDATIVE_PHOSPHORYLATION | KIF26B-AS1 |
| HALLMARK_OXIDATIVE_PHOSPHORYLATION | KIRREL3-AS2 |
| HALLMARK_OXIDATIVE_PHOSPHORYLATION | LINC00226 |
| HALLMARK_OXIDATIVE_PHOSPHORYLATION | LINC00350 |
| HALLMARK_INTERFERON_GAMMA_RESPONSE | LINC00355 |
| HALLMARK_INTERFERON_ALPHA_RESPONSE | LINC00355 |
| HALLMARK_MYC_TARGETS_V1 | LINC00355 |
| HALLMARK_OXIDATIVE_PHOSPHORYLATION | LINC00355 |
| HALLMARK_INFLAMMATORY_RESPONSE | LINC00374 |
| HALLMARK_INFLAMMATORY_RESPONSE | LINC00374 |
| HALLMARK_OXIDATIVE_PHOSPHORYLATION | LINC00443 |
| HALLMARK_MTORC1_SIGNALING | LINC00443 |
| HALLMARK_INFLAMMATORY_RESPONSE | LINC00964 |
| HALLMARK_INFLAMMATORY_RESPONSE | LINC00964 |
| HALLMARK_INFLAMMATORY_RESPONSE | LINC00974 |
| HALLMARK_INTERFERON_GAMMA_RESPONSE | LINC00992 |
| HALLMARK_INTERFERON_ALPHA_RESPONSE | LINC00992 |
| HALLMARK_MYC_TARGETS_V1 | LINC00992 |
| HALLMARK_OXIDATIVE_PHOSPHORYLATION | LINC00992 |
| HALLMARK_IL6_JAK_STAT3_SIGNALING | LINC00992 |
| HALLMARK_INFLAMMATORY_RESPONSE | LINC01067 |
| HALLMARK_INFLAMMATORY_RESPONSE | LINC01067 |
| HALLMARK_INTERFERON_GAMMA_RESPONSE | LINC01067 |
| HALLMARK_INTERFERON_GAMMA_RESPONSE | LINC01067 |
| HALLMARK_INTERFERON_GAMMA_RESPONSE | LINC01094 |
| HALLMARK_INTERFERON_ALPHA_RESPONSE | LINC01094 |
| HALLMARK_MYC_TARGETS_V1 | LINC01094 |
| HALLMARK_OXIDATIVE_PHOSPHORYLATION | LINC01094 |
| HALLMARK_MTORC1_SIGNALING | LINC01201 |
| HALLMARK_OXIDATIVE_PHOSPHORYLATION | LINC01201 |
| HALLMARK_OXIDATIVE_PHOSPHORYLATION | LINC01506 |
| HALLMARK_MTORC1_SIGNALING | LINC01506 |
| HALLMARK_OXIDATIVE_PHOSPHORYLATION | LINC01646 |
| HALLMARK_INTERFERON_GAMMA_RESPONSE | LINC01701 |
| HALLMARK_INTERFERON_ALPHA_RESPONSE | LINC01701 |
| HALLMARK_MYC_TARGETS_V1 | LINC01701 |
| HALLMARK_OXIDATIVE_PHOSPHORYLATION | LINC01701 |
| HALLMARK_INTERFERON_GAMMA_RESPONSE | LINC01765 |
| HALLMARK_INTERFERON_ALPHA_RESPONSE | LINC01765 |
| HALLMARK_MYC_TARGETS_V1 | LINC01765 |
| HALLMARK_OXIDATIVE_PHOSPHORYLATION | LINC01765 |
| HALLMARK_IL6_JAK_STAT3_SIGNALING | LINC01765 |
| HALLMARK_INTERFERON_GAMMA_RESPONSE | LINC01767 |
| HALLMARK_INTERFERON_ALPHA_RESPONSE | LINC01767 |
| HALLMARK_MYC_TARGETS_V1 | LINC01767 |
| HALLMARK_OXIDATIVE_PHOSPHORYLATION | LINC01767 |
| HALLMARK_IL6_JAK_STAT3_SIGNALING | LINC01767 |
| HALLMARK_INTERFERON_GAMMA_RESPONSE | LINC01861 |
| HALLMARK_INTERFERON_ALPHA_RESPONSE | LINC01861 |
| HALLMARK_MYC_TARGETS_V1 | LINC01861 |
| HALLMARK_OXIDATIVE_PHOSPHORYLATION | LINC01861 |
| HALLMARK_IL6_JAK_STAT3_SIGNALING | LINC01861 |
| HALLMARK_TNFA_SIGNALING_VIA_NFKB | LINC01986 |
| HALLMARK_HYPOXIA | LINC01986 |
| HALLMARK_UNFOLDED_PROTEIN_RESPONSE | LINC01986 |
| HALLMARK_INTERFERON_GAMMA_RESPONSE | LINC01994 |
| HALLMARK_INTERFERON_ALPHA_RESPONSE | LINC01994 |
| HALLMARK_MYC_TARGETS_V1 | LINC01994 |
| HALLMARK_OXIDATIVE_PHOSPHORYLATION | LINC01994 |
| HALLMARK_OXIDATIVE_PHOSPHORYLATION | LINC02050 |
| HALLMARK_MTORC1_SIGNALING | LINC02050 |
| HALLMARK_TNFA_SIGNALING_VIA_NFKB | LINC02273 |
| HALLMARK_APOPTOSIS | LINC02273 |
| HALLMARK_INTERFERON_GAMMA_RESPONSE | LINC02273 |
| HALLMARK_MYC_TARGETS_V1 | LINC02273 |
| HALLMARK_INFLAMMATORY_RESPONSE | LINC02273 |
| HALLMARK_IL2_STAT5_SIGNALING | LINC02273 |
| HALLMARK_INFLAMMATORY_RESPONSE | LINC02318 |
| HALLMARK_INFLAMMATORY_RESPONSE | LINC02318 |
| HALLMARK_INTERFERON_GAMMA_RESPONSE | LINC02345 |
| HALLMARK_INTERFERON_ALPHA_RESPONSE | LINC02345 |
| HALLMARK_MYC_TARGETS_V1 | LINC02345 |
| HALLMARK_OXIDATIVE_PHOSPHORYLATION | LINC02345 |
| HALLMARK_INFLAMMATORY_RESPONSE | LINC02386 |
| HALLMARK_INFLAMMATORY_RESPONSE | LINC02386 |
| HALLMARK_INTERFERON_GAMMA_RESPONSE | LINC02386 |
| HALLMARK_INTERFERON_GAMMA_RESPONSE | LINC02386 |
| HALLMARK_INFLAMMATORY_RESPONSE | LINC02436 |
| HALLMARK_OXIDATIVE_PHOSPHORYLATION | LINC02450 |
| HALLMARK_INFLAMMATORY_RESPONSE | LINC02497 |
| HALLMARK_INFLAMMATORY_RESPONSE | LINC02497 |
| HALLMARK_INTERFERON_GAMMA_RESPONSE | LINC02500 |
| HALLMARK_INTERFERON_ALPHA_RESPONSE | LINC02500 |
| HALLMARK_MYC_TARGETS_V1 | LINC02500 |
| HALLMARK_OXIDATIVE_PHOSPHORYLATION | LINC02500 |
| HALLMARK_INFLAMMATORY_RESPONSE | LINC02611 |
| HALLMARK_TNFA_SIGNALING_VIA_NFKB | LINC02611 |
| HALLMARK_INTERFERON_GAMMA_RESPONSE | LINC02611 |
| HALLMARK_INFLAMMATORY_RESPONSE | LINC02632 |
| HALLMARK_KRAS_SIGNALING_UP | LINC02632 |
| HALLMARK_SPERMATOGENESIS | LINC02632 |
| HALLMARK_TNFA_SIGNALING_VIA_NFKB | LINC02632 |
| HALLMARK_INFLAMMATORY_RESPONSE | LINC02650 |
| HALLMARK_INTERFERON_GAMMA_RESPONSE | LINC02650 |
| HALLMARK_OXIDATIVE_PHOSPHORYLATION | LINC02819 |
| HALLMARK_MYC_TARGETS_V1 | LINC02827 |
| HALLMARK_TNFA_SIGNALING_VIA_NFKB | LINC02827 |
| HALLMARK_INTERFERON_GAMMA_RESPONSE | LINCR-0001 |
| HALLMARK_INTERFERON_ALPHA_RESPONSE | LINCR-0001 |
| HALLMARK_MYC_TARGETS_V1 | LINCR-0001 |
| HALLMARK_OXIDATIVE_PHOSPHORYLATION | LINCR-0001 |
| HALLMARK_IL6_JAK_STAT3_SIGNALING | LINCR-0001 |
| HALLMARK_INTERFERON_GAMMA_RESPONSE | MACROD2-IT1 |
| HALLMARK_INTERFERON_ALPHA_RESPONSE | MACROD2-IT1 |
| HALLMARK_MYC_TARGETS_V1 | MACROD2-IT1 |
| HALLMARK_OXIDATIVE_PHOSPHORYLATION | MACROD2-IT1 |
| HALLMARK_IL6_JAK_STAT3_SIGNALING | MACROD2-IT1 |
| HALLMARK_TNFA_SIGNALING_VIA_NFKB | MAPK10-AS1 |
| HALLMARK_APOPTOSIS | MAPK10-AS1 |
| HALLMARK_INTERFERON_GAMMA_RESPONSE | MAPK10-AS1 |
| HALLMARK_INFLAMMATORY_RESPONSE | MAPK10-AS1 |
| HALLMARK_MYC_TARGETS_V1 | MAPK10-AS1 |
| HALLMARK_IL2_STAT5_SIGNALING | MAPK10-AS1 |
| HALLMARK_INFLAMMATORY_RESPONSE | MECOM-AS1 |
| HALLMARK_TNFA_SIGNALING_VIA_NFKB | MMP2-AS1 |
| HALLMARK_APOPTOSIS | MMP2-AS1 |
| HALLMARK_INTERFERON_GAMMA_RESPONSE | MMP2-AS1 |
| HALLMARK_INFLAMMATORY_RESPONSE | MMP2-AS1 |
| HALLMARK_MYC_TARGETS_V1 | MMP2-AS1 |
| HALLMARK_IL2_STAT5_SIGNALING | MMP2-AS1 |
| HALLMARK_INTERFERON_GAMMA_RESPONSE | MYLK-AS1 |
| HALLMARK_INTERFERON_ALPHA_RESPONSE | MYLK-AS1 |
| HALLMARK_MYC_TARGETS_V1 | MYLK-AS1 |
| HALLMARK_OXIDATIVE_PHOSPHORYLATION | MYLK-AS1 |
| HALLMARK_IL6_JAK_STAT3_SIGNALING | MYLK-AS1 |
| HALLMARK_OXIDATIVE_PHOSPHORYLATION | MZF1-AS1 |
| HALLMARK_INFLAMMATORY_RESPONSE | NANOGP11 |
| HALLMARK_TNFA_SIGNALING_VIA_NFKB | NANOGP11 |
| HALLMARK_KRAS_SIGNALING_UP | NANOGP11 |
| HALLMARK_SPERMATOGENESIS | NANOGP11 |
| HALLMARK_INTERFERON_GAMMA_RESPONSE | NINJ2-AS1 |
| HALLMARK_INTERFERON_ALPHA_RESPONSE | NINJ2-AS1 |
| HALLMARK_MYC_TARGETS_V1 | NINJ2-AS1 |
| HALLMARK_OXIDATIVE_PHOSPHORYLATION | NINJ2-AS1 |
| HALLMARK_OXIDATIVE_PHOSPHORYLATION | RERG-IT1 |
| HALLMARK_OXIDATIVE_PHOSPHORYLATION | RHOA-IT1 |
| HALLMARK_TNFA_SIGNALING_VIA_NFKB | RIPOR3-AS1 |
| HALLMARK_APOPTOSIS | RIPOR3-AS1 |
| HALLMARK_INTERFERON_GAMMA_RESPONSE | RIPOR3-AS1 |
| HALLMARK_INFLAMMATORY_RESPONSE | RIPOR3-AS1 |
| HALLMARK_MYC_TARGETS_V1 | RIPOR3-AS1 |
| HALLMARK_IL2_STAT5_SIGNALING | RIPOR3-AS1 |
| HALLMARK_INTERFERON_GAMMA_RESPONSE | RNASEH1-AS1 |
| HALLMARK_INTERFERON_ALPHA_RESPONSE | RNASEH1-AS1 |
| HALLMARK_MYC_TARGETS_V1 | RNASEH1-AS1 |
| HALLMARK_OXIDATIVE_PHOSPHORYLATION | RNASEH1-AS1 |
| HALLMARK_IL6_JAK_STAT3_SIGNALING | RNASEH1-AS1 |
| HALLMARK_OXIDATIVE_PHOSPHORYLATION | SH3PXD2A-AS1 |
| HALLMARK_INTERFERON_GAMMA_RESPONSE | SND1-IT1 |
| HALLMARK_INTERFERON_ALPHA_RESPONSE | SND1-IT1 |
| HALLMARK_MYC_TARGETS_V1 | SND1-IT1 |
| HALLMARK_OXIDATIVE_PHOSPHORYLATION | SND1-IT1 |
| HALLMARK_INTERFERON_GAMMA_RESPONSE | SYNJ2-IT1 |
| HALLMARK_INTERFERON_ALPHA_RESPONSE | SYNJ2-IT1 |
| HALLMARK_MYC_TARGETS_V1 | SYNJ2-IT1 |
| HALLMARK_OXIDATIVE_PHOSPHORYLATION | SYNJ2-IT1 |
| HALLMARK_IL6_JAK_STAT3_SIGNALING | SYNJ2-IT1 |
| HALLMARK_INFLAMMATORY_RESPONSE | WASF3-AS1 |
| HALLMARK_OXIDATIVE_PHOSPHORYLATION | Z93930.2 |
| HALLMARK_OXIDATIVE_PHOSPHORYLATION | Z97192.4 |
| HALLMARK_INFLAMMATORY_RESPONSE | ZNF197-AS1 |
| HALLMARK_INFLAMMATORY_RESPONSE | ZNF197-AS1 |
| HALLMARK_INFLAMMATORY_RESPONSE | ZNF341-AS1 |
| HALLMARK_INFLAMMATORY_RESPONSE | ZNF341-AS1 |
