# Supplementary material for: Identification of Immune-Related lncRNA Regulatory Network in Pulpitis
Source: Dis Markers. 2022 Jun 6;2022:7222092. doi: 10.1155/2022/7222092 (PMC9194960; doi:10.1155/2022/7222092)
Supplement: Supplementary 9 — Table S4: infiltrating immunocytes scores between two immune subgroups. [file 7222092.f9.docx]

| Table S4 Infiltrating immunocytes scores between two immune subgroups |
| --- |

| cell_category | GEO_accession | score | group |
| --- | --- | --- | --- |
| T cells | GSM2434474 | 0.374190427 | sub1 |
| CD8 T cells | GSM2434474 | 0.1024294 | sub1 |
| Cytotoxic lymphocytes | GSM2434474 | 0.294626824 | sub1 |
| B lineage | GSM2434474 | 0.326346395 | sub1 |
| NK cells | GSM2434474 | 0.357345685 | sub1 |
| Monocytic lineage | GSM2434474 | 0.583975655 | sub1 |
| Myeloid dendritic cells | GSM2434474 | 0.644019406 | sub1 |
| Neutrophils | GSM2434474 | 0.503983589 | sub1 |
| Endothelial cells | GSM2434474 | 0.622135343 | sub1 |
| Fibroblasts | GSM2434474 | 0.933379522 | sub1 |
| T cells | GSM2434476 | 0.368964586 | sub1 |
| CD8 T cells | GSM2434476 | 0.085025398 | sub1 |
| Cytotoxic lymphocytes | GSM2434476 | 0.327948295 | sub1 |
| B lineage | GSM2434476 | 0.310737538 | sub1 |
| NK cells | GSM2434476 | 0.346794108 | sub1 |
| Monocytic lineage | GSM2434476 | 0.550948509 | sub1 |
| Myeloid dendritic cells | GSM2434476 | 0.697796983 | sub1 |
| Neutrophils | GSM2434476 | 0.482605721 | sub1 |
| Endothelial cells | GSM2434476 | 0.659760395 | sub1 |
| Fibroblasts | GSM2434476 | 0.94130289 | sub1 |
| T cells | GSM2434477 | 0.308049949 | sub2 |
| CD8 T cells | GSM2434477 | 0 | sub2 |
| Cytotoxic lymphocytes | GSM2434477 | 0.251107036 | sub2 |
| B lineage | GSM2434477 | 0.235852049 | sub2 |
| NK cells | GSM2434477 | 0.318019733 | sub2 |
| Monocytic lineage | GSM2434477 | 0.562103423 | sub2 |
| Myeloid dendritic cells | GSM2434477 | 0.637434168 | sub2 |
| Neutrophils | GSM2434477 | 0.5679641 | sub2 |
| Endothelial cells | GSM2434477 | 0.659797657 | sub2 |
| Fibroblasts | GSM2434477 | 1 | sub2 |
| T cells | GSM2434478 | 0.280324427 | sub2 |
| CD8 T cells | GSM2434478 | 0.001857602 | sub2 |
| Cytotoxic lymphocytes | GSM2434478 | 0.273115843 | sub2 |
| B lineage | GSM2434478 | 0.230564887 | sub2 |
| NK cells | GSM2434478 | 0.308295498 | sub2 |
| Monocytic lineage | GSM2434478 | 0.528063721 | sub2 |
| Myeloid dendritic cells | GSM2434478 | 0.619472851 | sub2 |
| Neutrophils | GSM2434478 | 0.566471363 | sub2 |
| Endothelial cells | GSM2434478 | 0.637916073 | sub2 |
| Fibroblasts | GSM2434478 | 0.969226766 | sub2 |
| T cells | GSM2434479 | 0.286642393 | sub2 |
| CD8 T cells | GSM2434479 | 0.016940754 | sub2 |
| Cytotoxic lymphocytes | GSM2434479 | 0.239417625 | sub2 |
| B lineage | GSM2434479 | 0.214406455 | sub2 |
| NK cells | GSM2434479 | 0.326644752 | sub2 |
| Monocytic lineage | GSM2434479 | 0.572488153 | sub2 |
| Myeloid dendritic cells | GSM2434479 | 0.63316356 | sub2 |
| Neutrophils | GSM2434479 | 0.602455384 | sub2 |
| Endothelial cells | GSM2434479 | 0.634813121 | sub2 |
| Fibroblasts | GSM2434479 | 0.979589177 | sub2 |
