# Supplementary material for: Identification of Immune-Related lncRNA Regulatory Network in Pulpitis
Source: Dis Markers. 2022 Jun 6;2022:7222092. doi: 10.1155/2022/7222092 (PMC9194960; doi:10.1155/2022/7222092)
Supplement: Supplementary 10 — Table S5: relative pathway scores between two immune subgroups. [file 7222092.f10.docx]

Table S5 Relative pathway scores between two immune subgroups

| immunological_category | GEO_accession | | score | group |
| --- | --- | --- | --- | --- |
| Antigen_Processing_and_  Presentation | GSM2434474 | 0.134068589 | | sub1 |
| Antimicrobials | GSM2434474 | -0.140475772 | | sub1 |
| BCR_Signaling_Pathway | GSM2434474 | 0.240705744 | | sub1 |
| Chemokine_Receptors | GSM2434474 | 0.247783758 | | sub1 |
| Chemokines | GSM2434474 | -0.277953313 | | sub1 |
| Cytokine_Receptors | GSM2434474 | 0.189632133 | | sub1 |
| Cytokines | GSM2434474 | -0.191757025 | | sub1 |
| Interferons | GSM2434474 | -0.479841569 | | sub1 |
| Interferons_Receptors | GSM2434474 | 0.858367412 | | sub1 |
| Interleukins | GSM2434474 | -0.237709123 | | sub1 |
| Interleukins_Receptors | GSM2434474 | 0.176146366 | | sub1 |
| Natural_Killer_Cell | GSM2434474 | -0.169097898 | | sub1 |
| TCR_Signaling_Pathway | GSM2434474 | 0.290019244 | | sub1 |
| TGF-b_Family_Members | GSM2434474 | 0.227349939 | | sub1 |
| TGF-b_Family_Members_Receptors | GSM2434474 | 0.505588964 | | sub1 |
| TNF_Family_Members | GSM2434474 | 0.281573003 | | sub1 |
| TNF_Family_Members_Receptors | GSM2434474 | -0.261831588 | | sub1 |
| Antigen_Processing_and_  Presentation | GSM2434476 | 0.165707539 | | sub1 |
| Antimicrobials | GSM2434476 | 0.123785472 | | sub1 |
| BCR_Signaling_Pathway | GSM2434476 | 0.189899133 | | sub1 |
| Chemokine_Receptors | GSM2434476 | 0.259645415 | | sub1 |
| Chemokines | GSM2434476 | 0.181152839 | | sub1 |
| Cytokine_Receptors | GSM2434476 | 0.185070985 | | sub1 |
| Cytokines | GSM2434476 | 0.246959488 | | sub1 |
| Interferons | GSM2434476 | -0.399418954 | | sub1 |
| Interferons_Receptors | GSM2434476 | 0.392401225 | | sub1 |
| Interleukins | GSM2434476 | 0.263335271 | | sub1 |
| Interleukins_Receptors | GSM2434476 | 0.307594645 | | sub1 |
| Natural_Killer_Cell | GSM2434476 | 0.136585342 | | sub1 |
| TCR_Signaling_Pathway | GSM2434476 | 0.220948345 | | sub1 |
| TGF-b_Family_Members | GSM2434476 | 0.170752533 | | sub1 |
| TGF-b Family_Members_Receptors | GSM2434476 | -0.461498415 | | sub1 |
| TNF_Family_Members | GSM2434476 | 0.323981143 | | sub1 |
| TNF_Family_Members_Receptors | GSM2434476 | 0.22782069 | | sub1 |
| Antigen_Processing_and_  Presentation | GSM2434477 | 0.229011067 | | sub2 |
| Antimicrobials | GSM2434477 | 0.197579744 | | sub2 |
| BCR_Signaling_Pathway | GSM2434477 | 0.291087548 | | sub2 |
| Chemokine_Receptors | GSM2434477 | -0.235494599 | | sub2 |
| Chemokines | GSM2434477 | 0.33603422 | | sub2 |
| Cytokine_Receptors | GSM2434477 | -0.151876769 | | sub2 |
| Cytokines | GSM2434477 | 0.183772695 | | sub2 |
| Interferons | GSM2434477 | 0.316765003 | | sub2 |
| Interferons_Receptors | GSM2434477 | -0.430704212 | | sub2 |
| Interleukins | GSM2434477 | -0.250961463 | | sub2 |
| Interleukins_Receptors | GSM2434477 | -0.197205891 | | sub2 |
| Natural_Killer_Cell | GSM2434477 | 0.246792912 | | sub2 |
| TCR_Signaling_Pathway | GSM2434477 | 0.205031143 | | sub2 |
| TGF-b_Family_Members | GSM2434477 | 0.351556844 | | sub2 |
| TGF-b_Family_Members_Receptors | GSM2434477 | 0.19399407 | | sub2 |
| TNF_Family_Members | GSM2434477 | 0.26011364 | | sub2 |
| TNF_Family_Members_Receptors | GSM2434477 | 0.411121243 | | sub2 |
| Antigen_Processing_and_  Presentation | GSM2434478 | -0.301746979 | | sub2 |
| Antimicrobials | GSM2434478 | -0.240920814 | | sub2 |
| BCR_Signaling_Pathway | GSM2434478 | -0.325723353 | | sub2 |
| Chemokine_Receptors | GSM2434478 | -0.383520766 | | sub2 |
| Chemokines | GSM2434478 | -0.217727003 | | sub2 |
| Cytokine_Receptors | GSM2434478 | -0.230293967 | | sub2 |
| Cytokines | GSM2434478 | -0.243093386 | | sub2 |
| Interferons | GSM2434478 | 0.256433764 | | sub2 |
| Interferons_Receptors | GSM2434478 | -0.487101964 | | sub2 |
| Interleukins | GSM2434478 | -0.355499675 | | sub2 |
| Interleukins_Receptors | GSM2434478 | -0.372563937 | | sub2 |
| Natural_Killer_Cell | GSM2434478 | -0.292054349 | | sub2 |
| TCR_Signaling_Pathway | GSM2434478 | -0.319425204 | | sub2 |
| TGF-b_Family_Members | GSM2434478 | -0.36720425 | | sub2 |
| TGF-b_Family_Members_Receptors | GSM2434478 | 0.293659308 | | sub2 |
| TNF_Family_Members | GSM2434478 | -0.46647692 | | sub2 |
| TNF_Family_Members_Receptors | GSM2434478 | -0.315074816 | | sub2 |
| Antigen_Processing_and_  Presentation | GSM2434479 | 0.23327897 | | sub2 |
| Antimicrobials | GSM2434479 | 0.226424649 | | sub2 |
| BCR_Signaling_Pathway | GSM2434479 | -0.214357868 | | sub2 |
| Chemokine_Receptors | GSM2434479 | 0.258678898 | | sub2 |
| Chemokines | GSM2434479 | 0.206063076 | | sub2 |
| Cytokine_Receptors | GSM2434479 | 0.136685099 | | sub2 |
| Cytokines | GSM2434479 | 0.169083487 | | sub2 |
| Interferons | GSM2434479 | 0.595701691 | | sub2 |
| Interferons_Receptors | GSM2434479 | -0.500162206 | | sub2 |
| Interleukins | GSM2434479 | 0.313901513 | | sub2 |
| Interleukins_Receptors | GSM2434479 | 0.326476859 | | sub2 |
| Natural_Killer_Cell | GSM2434479 | 0.243839923 | | sub2 |
| TCR_Signaling_Pathway | GSM2434479 | -0.198779509 | | sub2 |
| TGF-b_Family_Members | GSM2434479 | -0.284896235 | | sub2 |
| TGF-b_Family_Members_Receptors | GSM2434479 | 0.208269933 | | sub2 |
| TNF_Family_Members | GSM2434479 | -0.38393835 | | sub2 |
| TNF_Family_Members_Receptors | GSM2434479 | 0.208431556 | | sub2 |
